# Supplementary material for: What are older smokers’ attitudes to quitting and how are they managed in primary care? An analysis of the cross-sectional English Smoking Toolkit Study
Source: BMJ Open. 2017 Nov 15;7(11):e018150. doi: 10.1136/bmjopen-2017-018150 (PMC5695521; doi:10.1136/bmjopen-2017-018150)
Supplement: Supplementary data [file bmjopen-2017-018150supp001.pdf]

Supplemental Tables

Supplemental Table 1: Descriptive statistics of sociodemographics and time period with smoking status, past quitting and use of cessation therapies or GP management

		Ever Smoker	Nicotine Dependency	Quit attempts in past year	Past NRT use	Past NHS cessation clinics use	Respondent raised the topic of smoking with GP	GP spoke to respondent about quitting smoking	GP referrals to smoking cessation therapies
		N=68,967	N=51,920	N=43,385	N=20,286	N=16,026	N=11,432	N=29,578	N=10,466
**Age**		%	%	%	%	%	%	%	%
	**16-54**	61.4	69.4	74.6	75.8	80.1	73.6	73.7	69.2
	**55-59**	7.0	6.8	6.6	6.9	5.8	6.7	6.8	7.5
	**60-64**	8.2	7.2	6.4	6.4	5.4	6.4	6.6	7.6
	**65-69**	7.7	6.1	5.1	5.0	4.1	5.8	5.4	6.6
	**70-74**	6.2	4.5	3.6	3.1	2.6	3.6	3.8	4.6
	**75-79**	4.7	3.2	2.2	1.7	1.2	2.1	2.2	2.7
	**80-84**	3.0	1.8	1.1	0.7	0.4	1.0	1.1	1.2
	**85+**	1.9	0.9	0.5	0.3	0.2	0.7	0.6	0.7
	**Total**	100.0	100.0	100.0	100.0	100.0	100.0	100.0	100.0
**Gender**		%	%	%	%	%	%	%	%
	**Men**	48.0	51.0	50.5	48.4	47.2	54.1	52.0	47.4
	**Women**	52.0	49.0	49.5	51.6	52.8	46.0	48.0	52.7
	**Total**	100.0	100.0	100.0	100.0	100.0	100.0	100.0	100.0
**Social Grade**		%	%	%	%	%	%	%	%
	**AB**	15.2	12.4	10.5	10.6	10.9	10.8	10.6	10.5
	**C1**	24.9	23.8	22.9	22.9	23.7	24.8	22.8	21.8
	**C2**	22.2	22.5	22.7	22.6	22.7	23.7	22.4	21.5
	**D**	16.9	18.3	19.3	18.8	18.9	20.0	19.4	18.9
	**E**	20.8	23.1	24.6	25.1	23.8	20.7	24.8	27.3
	**Total**	100.0	100.0	100.0	100.0	100.0	100.0	100.0	100.0
**Survey Year**		%	%	%	%	%	%	%	%
	**2006**	2.1	2.8	2.3	2.6	2.8			
	**2007**	13.3	17.6	14.4	16.2	16.5			
	**2008**	9.9	13.1	11.1	12.4	11.8			
	**2009**	10.5	13.9	11.9	13.1	11.8		11.7	
	**2010**	13.7	13.2	14.0	14.6	13.4		20.2	21.9
	**2011**	12.5	10.2	12.2	12.6	10.8		17.1	19.0
	**2012**	11.9	9.9	11.9	8.1	10.8	12.0	17.0	20.0
	**2013**	12.7	9.3	10.7	9.2	11.0	42.2	16.3	18.4
	**2014**	10.8	8.2	9.4	9.2	9.4	37.1	14.3	16.7
	**2015**	2.8	1.9	2.2	2.0	1.8	8.7	3.3	4.1
	**Total**	100.0	100.0	100.0	100.0	100.0	100.0	100.0	100.0

Supplemental Table 2: Odds ratios for sociodemographics and time period with past use of NRTs or stop smoking counselling services

		**Past NRT use**					**Past NHS advice services use**			
		**OR**	**P-value**	**95% CI**			**OR**	**P-value**	**95% CI**	
**Age**										
	**16-54**	1					1			
	**55-64**	1.132	0.012	1.028	1.247		1.555	<0.001	1.303	1.856
	**65-74**	1.086	0.182	0.962	1.227		1.378	0.007	1.089	1.742
	**75+**	0.703	0.003	0.559	0.886		0.994	0.981	0.613	1.613
**Gender**										
	**Men**	0.903	0.003	0.845	0.966		0.773	<0.001	0.680	0.879
	**Women**	1					1			
**Social Grade**										
	**AB**	1					1			
	**C1**	0.884	0.047	0.783	0.999		0.920	0.478	0.731	1.158
	**C2**	0.887	0.054	0.785	1.002		0.890	0.327	0.706	1.123
	**D**	0.801	0.001	0.705	0.909		1.059	0.630	0.838	1.338
	**E**	0.899	0.081	0.797	1.013		0.973	0.815	0.776	1.221
**Survey Years**										
	**2006-08**	1					1			
	**2009-10**	1.065	0.138	0.980	1.158		0.885	0.122	0.758	1.033
	**2011-12**	0.839	<0.001	0.764	0.922		0.612	<0.001	0.512	0.733
	**2013-15**	0.597	<0.001	0.540	0.661		0.495	<0.001	0.409	0.599

Supplemental Table 3: Odds ratios for sociodemographics with different GP management compared to no referral

		**GP referrals to help quit smoking**															
		**Counselling vs. No Referral**				**Pharmacology vs. No Referral**				**Counselling and Pharmacology vs. No Referral**				**GP did not advise to quit smoking vs. No Referral**			
		**OR**	**P-value**	**95% CI**		**OR**	**P-value**	**95% CI**		**OR**	**P-value**	**95% CI**		**OR**	**P-value**	**95% CI**	
**Age**																	
	**16-54**	1				1				1				1			
	**55-59**	0.869	0.126	0.726	1.040	0.814	0.068	0.653	1.016	1.331	0.125	0.924	1.916	1.102	0.428	0.866	1.403
	**60-64**	0.944	0.515	0.794	1.123	0.864	0.179	0.697	1.069	1.821	<0.001	1.318	2.515	1.097	0.443	0.866	1.389
	**65-69**	1.034	0.734	0.851	1.258	1.077	0.530	0.855	1.356	2.127	<0.001	1.497	3.024	1.391	0.010	1.081	1.789
	**70-74**	0.690	0.002	0.547	0.869	0.854	0.244	0.654	1.114	1.390	0.137	0.900	2.146	1.476	0.004	1.128	1.930
	**75-79**	0.512	<0.001	0.377	0.696	0.551	0.002	0.380	0.799	0.618	0.201	0.296	1.291	1.636	0.002	1.190	2.248
	**80+**	0.361	<0.001	0.241	0.539	0.251	<0.001	0.138	0.456	0.109	0.028	0.015	0.787	1.309	0.191	0.875	1.959
**Gender**																	
	**Men**	0.820	<0.001	0.744	0.904	0.991	0.886	0.882	1.115	0.798	0.034	0.647	0.983	0.960	0.547	0.842	1.095
	**Women**	1				1				1				1			
**Social Grade**																	
	**AB**	1				1				1				1			
	**C1**	1.064	0.510	0.884	1.280	1.019	0.874	0.810	1.282	0.995	0.979	0.691	1.434	0.946	0.651	0.744	1.203
	**C2**	1.182	0.077	0.982	1.422	1.225	0.080	0.976	1.536	0.697	0.068	0.473	1.027	0.894	0.366	0.701	1.140
	**D**	1.129	0.208	0.935	1.363	1.184	0.153	0.939	1.491	0.728	0.112	0.492	1.077	0.908	0.444	0.708	1.163
	**E**	1.214	0.033	1.016	1.451	1.311	0.015	1.054	1.631	0.860	0.404	0.603	1.226	0.978	0.849	0.776	1.232
**Survey Years**																	
	**2009-10**	1				1				1				1			
	**2011-12**	0.899	0.101	0.792	1.021	1.325	<0.001	1.137	1.543	0.808	0.087	0.633	1.031	0.847	0.060	0.712	1.007
	**2013-15**	0.899	0.103	0.791	1.022	0.880	0.120	0.750	1.034	0.408	<0.001	0.307	0.540	0.983	0.844	0.829	1.166

Supplemental table 4: sensitivity analysis for association between age and whether the doctor raised smoking or offered any active management to quit

		GP offered any active management to quit								GP raised smoking							
		Original Model (sample from w40-102) (n=9,154)				Model with sample from w72-102 (n=3,800)				Original Model (sample from w40-102) (n=29,578)				Model with sample from w72-102 (n=11,425)			
		**OR**	**P-value**	**95% CI**		**OR**	**P-value**	**95% CI**		**OR**	**P-value**	**95% CI**		**OR**	**P-value**	**95% CI**	
**Age**																	
	**16-54**	1				1				1				1			
	**55-59**	1.14	0.126	0.97	1.34	1.06	0.675	0.82	1.36	1.58	0	1.44	1.74	1.65	0	1.43	1.93
	**60-64**	1.04	0.636	0.89	1.22	1.07	0.554	0.84	1.36	1.88	0	1.72	2.07	2.32	0	1.99	2.72
	**65-69**	0.90	0.233	0.75	1.07	0.85	0.208	0.66	1.10	1.98	0	1.78	2.19	2.38	0	2.02	2.80
	**70-74**	1.27	0	1.03	1.55	1.13	0.451	0.82	1.55	1.85	0	1.63	2.09	2.08	0	1.71	2.55
	**75-79**	1.87	0	1.43	2.44	1.82	0.006	1.19	2.79	1.69	0	1.44	1.98	1.78	0	1.37	2.30
	**80+**	3.16	0.002	2.20	1.55	2.34	0.002	1.38	3.98	1.02	0.804	0.85	1.24	1.30	0.08	0.97	1.74
