# Supplementary material for: What are older smokers’ attitudes to quitting and how are they managed in primary care? An analysis of the cross-sectional English Smoking Toolkit Study
Source: BMJ Open. 2017 Nov 15;7(11):e018150. doi: 10.1136/bmjopen-2017-018150 (PMC5695521; doi:10.1136/bmjopen-2017-018150)
Supplement: Author's manuscript [file bmjopen-2017-018150.draft_revisions.pdf]

BMJ Open is committed to open peer review. As part of this commitment we make the peer review history of every article we publish publicly available.

When an article is published we post the peer reviewers' comments and the authors' responses online. We also post the versions of the paper that were used during peer review. These are the versions that the peer review comments apply to.

The versions of the paper that follow are the versions that were submitted during the peer review process. They are not the versions of record or the final published versions. They should not be cited or distributed as the published version of this manuscript.

BMJ Open is an open access journal and the full, final, typeset and author-corrected version of record of the manuscript is available on our site with no access controls, subscription charges or pay-per-view fees (http://bmjopen.bmj.com).

If you have any questions on BMJ Open's open peer review process please email editorial.bmjopen@bmj.com

BMJ Open

Older smokers' attitudes to quitting and how they are managed in primary care: An analysis of the English Smoking Toolkit Study

Journal:	*BMJ Open*
Manuscript ID	bmjopen-2017-018150
Article Type:	Research
Date Submitted by the Author:	13-Jun-2017
Complete List of Authors:	Jordan, Hannah; University of Sheffield, School of Health and Related Research<br>Hidajat, Mira; University of Bristol, School of Social and Community Medicine<br>Payne, Nick; University of Sheffield, School of Health and Related Research (ScHARR)<br>Adams, J; University of Cambridge, Centre for Diet & Activity Research<br>White, Martin; University of Cambridge School of Clinical Medicine, MRC Epidemiology Unit<br>Ben-Shlomo, Yoav; University of Bristol,
**Primary Subject Heading**:	Smoking and tobacco
Secondary Subject Heading:	Public health, General practice / Family practice, Health services research
Keywords:	PUBLIC HEALTH, Smoking cessation, Inequalities, Aging, PRIMARY CARE

SCHOLARONE™
Manuscripts

Older smokers' attitudes to quitting and how they are managed in primary care: An analysis of the English Smoking Toolkit Study

Hannah Jordan[1], Mira Hidajat[2], Nick Payne[1], Jean Adams[3], Martin White[3], Yoav Ben-Shlomo[2]

Affiliations

1. Section of Public Health, School of Health and Related Research (ScHARR), The University of Sheffield, Regent Court, 30 Regent Street, Sheffield S1 4DA
2. School of Social and Community Medicine, University of Bristol, 39 Whatley Road, Bristol BS8 2PS,
3. MRC Epidemiology Unit, University of Cambridge School of Clinical Medicine, Box 285 Institute of Metabolic Science, Cambridge Biomedical Campus, Cambridge, CB2 0QQ

HJ is the corresponding author (email: h.c.jordan@sheffield.ac.uk)

For peer review only

Abstract

Objectives

To investigate how age influenced access to smoking cessation services.

Design

Data from the Smoking Toolkit Study, a repeated multi-wave cross-sectional household survey conducted monthly from 2006 to 2015 (N=181,157).

Setting

England

Participants

Past-year smokers who participated in any of the 102 waves stratified into age groups.

Outcome measures

Amount smoked and nicotine dependency, self-reported quit attempts and use of smoking cessation interventions. Self-report of whether the GP raised the topic of smoking and made referrals for pharmacological support (NRTs) or other support (counselling or support groups).

Results

Older smokers (75 + years) were less likely to report that they were attempting to quit smoking or seek help from a GP, despite being less nicotine dependent. Doctors raised smoking as a topic equally across all age groups, but smokers aged 70 plus were more likely not to be referred for NRT or other support (odds ratios relative to 16-54 years; 70-74 years 1.27, 95%CI 1.03, 1.55; 75-79 1.87, 95% CI 1.43, 2.44; 80+ 3.16, 95% CI 2.20, 4.55; p-value for trend <0.001).

Conclusions

Our findings suggest that there are potential missed opportunities in facilitating smoking cessation in older smokers. In this large, population based study, older smokers appeared less interested in quitting and were less likely to be offered support, despite being less addicted to nicotine than younger smokers. It is unclear whether this constitutes inequitable access to

services or reflects informed choices by older smokers and their general practitioners. Future research is needed to understand why older smokers and GPs do not pursue smoking cessation. Service provision should consider how best to reduce these variations and a stronger effectiveness evidence base is required to support commissioning for this older population so that, where appropriate, older smokers are not missing out on smoking cessation therapies and the health benefits of cessation at older ages.

Strengths and limitations of this study

- The findings of the study are based on a large, representative survey of the adult population of England
- The large sample size enabled us to examine older age groups in far greater detail than in previous studies
- Quit behaviours and support were self-reported, raising questions of differences in recall or reporting behaviour among older smokers

For peer review only

Introduction

Despite a marked decline in smoking behaviour over the last few decades, around 10 million adults in the UK still smoke, of which 11% are over the age of 60[1]. There is, however, clear evidence for the benefits of quitting smoking at older ages. Large scale prospective cohort studies have found that smokers who quit after the age of 65 years benefit from reduced mortality (2-4 extra years of life)[2] additional healthy life years[3] and reduced morbidity[4]. Although the relative risks associated with smoking status decline with age, the absolute risk differences continue to increase[4].

The National Institute for Health and Care Excellence (NICE) recommends that smoking cessation advice should be provided to all smokers, regardless of age, including the oldest old. However, current policy aims to target specific groups, such as pregnant women and socio-economically disadvantaged populations, for cessation advice whilst older smokers are not generally recognised as a priority group[5]. A recent study reported that opportunities to offer cessation advice and support to older smokers may often be missed in Primary Care [6]. However, a recent review showed that pharmacotherapy such as Nicotine Replacement Therapy (NRT) was effective in older adults, but that the literature was sparse and effective smoking cessation services may need to be tailored to the needs of older adults[7].

There are several possible reasons why smoking cessation therapies may not be used equally across age-groups. It has been suggested that older smokers may be more strongly addicted to nicotine, which may hamper attempts to initiate quitting[8]. Older people themselves may have beliefs about quitting, considering themselves 'survivors', or believing that 'the damage is done' so that they see no point in attempting to quit later in life, resulting in reluctance to demand services or recognise the benefits of quitting[9]. Health professionals may have beliefs about older smokers that hinder their access to smoking cessation services, such as a reluctance to give cessation advice or to provide medication[10–13]. There may be structural factors such as the type, location and visibility of smoking cessation services which introduce barriers by age. For example, older smokers may be reluctant to use telephone or online support such as text messaging[14].

For peer review only

In this paper, we examine the potential reasons why older smokers may or may not have equitable access to smoking cessation therapies in a large, multi-wave, national cross-sectional survey in England. Because of the large sample size, we were able examine older age groups in far greater detail than in previous studies. We test the following hypotheses: Are older smokers compared to younger smokers as likely to: (a) attempt to quit or cut down on their cigarette consumption? (b) be nicotine dependent? (c) seek help from a general practitioner? In addition, are general practitioners as likely, when comparing older to younger smokers to: (d) discuss smoking cessation; and (e) refer patients to either smoking cessation services and/or prescribe NRTs?

Methods

Data

We used data from the Smoking Toolkit Study (STS), a repeated, multi-wave, cross-sectional household survey conducted approximately every month from November 2006 to January 2015 across England (102 waves of data, N=181,157)[15]. Each survey wave contains responses from approximately 1,800 adults aged 16+ (approximately 570 ex-smokers and 500 current smokers) collected during a face-to-face computer-assisted household survey. The sampling method is a hybrid between random probability and simple quota. The sample is identified by a random selection of over 170,000 localities ('output areas') after stratification by a geo-demographic analysis of the population. Each locality contains approximately 250 households. Interviewers visit households within the locality starting at a random point in the area. One member per household is interviewed until interviewers achieve local quotas designed to minimise differences in the probability of participation. The method has been shown to result in a sample that is nationally representative in its socio-demographic composition.[15]

Exposure

Our exposure of interest was age. We chose to operationalise age into bands, not assuming any linear relationship, as follows: 16 to 54 years as baseline then 5 year bands from 55 to 79, and 80+ as the oldest age-group.

Smoking status, nicotine dependency and reducing consumption

For peer review only

We classified smoking status into four categories: current smoker (respondents who indicated that they smoke cigarettes (including hand-rolled) or other forms of tobacco (e.g. pipe or cigar); recent smoker (respondents who indicated that they have stopped smoking within the past year); past smoker (respondents who indicated that they have not smoked for a year or more) and never smokers. For current, recent and past smokers, we categorized respondents into groups by the amount smoked: 10 or fewer cigarettes per day, 11-20 cigarettes per day, and 21 or more cigarettes per day.

Dependency and past use of services

Nicotine dependency (Yes/No) was classified using the Heaviness of Smoking Index (HSI)[16] , a validated index based on a subset of questions from the Fagerstrom Test of Nicotine Dependency[17]. (See box 1 for more details concerning specific outcomes).

We defined smoking cessation and reduction behaviours - quit attempts and cutting down - as dichotomous variables.

We classified whether current smokers used two types of smoking cessation interventions (Yes/No) - use of NRTs and NHS stop smoking counselling.

Doctor – patient interaction and management

The survey captures both patient and doctor initiated interactions around smoking cessation, allowing us to examine whether relevant respondents raised the topic of smoking with their GP and also whether the doctor raised the issue of smoking. For the latter question there were a series of outcome options such as a referral for stop smoking counselling, prescription of a NRT, both, or advice but no referral. To look at the determinants of advice only, we created a dichotomous variable by comparing no referral for counselling, prescription of NRT or both. Finally there was an option that the GP did not specifically advise the patient to stop smoking (no active management).

Potential confounders

We examined the following potential confounders: gender, secular period as measured by year of survey (two year bands) and a measure of socioeconomic status ('social grade' – see below). We argued that both social grade and gender could have influenced past smoking behaviour and may determine willingness to engage in cessation therapies as well as being

For peer review only

age-related (more women and more affluent older participants). Secular period may influence availability of cessation therapies.

Social grade was reported using the five occupation-based categories of the National Readership Survey method: AB (higher/intermediate managerial, administrative or professional), C1 (supervisory, clerical and junior managerial, administrative and professional), C2 (skilled manual workers), D (Semi-skilled and unskilled manual workers), and E (state pensioners, casual and lowest grade workers, unemployed with state benefits only[18]). The social grade of the household head was used to classify the entire household.

Statistical analyses

We conducted logistic regression analyses based on the relevant outcome variables (nicotine dependency, quit attempts, cutting down, past NRT usage, past NHS advice services used, patient or doctor raised smoking, GP referral for NRT, stop smoking counselling or just advice). We modelled age group both as a dummy variable and as an ordinal variable to test for a linear trend. Ordered logistic regression was performed for categories of amount smoked per day. To examine how management differed by age group we derived the odds ratios (ORs) (95% CI, p-value), using logistic regression models, for referral for counselling, NRT prescription, both or no active management, as compared to the baseline group of the GP giving advice to quit (advice only) but no referral. For each model we ran unadjusted and adjusted models conditioning on gender, social grade and secular period. Due to questionnaire changes, some questions were not available in all waves, particularly doctor-patient interactions, NHS stop smoking services use, and NRT use. In our analyses, we used the appropriate waves for each model accordingly although it meant using different waves of data across the various models (see Box 1). We conducted a sensitivity analysis using only the survey waves with complete data for GP-related questions (waves 72-102).

Results

Descriptive analyses

From a total sample of 181,157 participants, there were 41,031 (22.7%) current smokers, 2,825 (1.6%) recent smokers, 25,367 (14.0%) past smokers and 112,046 (61.9%) never smokers and 131 missing (0.07%). Supplemental Table 1 presents each outcome by socio-demographic characteristics and survey year. Older participants were less likely to be current

For peer review only

smokers, more likely to be former smokers and there was an inverted J shaped relationship for smoking intensity, with 55-59 year olds and those >65 years old less likely to be heavy smokers.

Nicotine dependency and quit attempts

Nicotine dependency showed an inverted J pattern so that participants aged 55 to 69 appeared to have higher nicotine dependency but from 70 years upwards there was a progressive reduction in the odds ratio for dependency (Table 2). However, older participants from 55 years onwards were less likely to report having at least one quit attempt. Men showed greater dependency and were less likely to quit. The odds of high nicotine dependency increased from higher to lower social grades and respondents from the lower social grades were also less likely to report a recent quit attempt. Participants from surveys in more recent years were less dependent and less likely to make quit attempts.

Past use of NRT or stop smoking counselling

Past use of NRTs was more common among 55 to 64 year olds compared to 16 to 54 year olds; use of stop smoking counselling was more common among 65 to 74 year olds. Older participants (75 + years) were less likely to have been prescribed NRT than 55-64 or 65-74 year olds (Figure 1). We found similar patterns by gender; men were less likely than women to have been prescribed NRTs or referred for counselling (Supplemental Table 2). Participants in social grade D were less likely to have been prescribed NRTs than those in social grade AB but there were no social grade differences in referral for counselling. There appeared to be a secular pattern so that respondents from recent survey years (2011-15) were less likely to have been prescribed NRTs and referred for counselling than those from 2006-08.

Raising smoking in consultations and management

We found an inverted J-shaped relationship for age group, such that older adults (75 years and above) were much less likely to have raised the topic of smoking during their GP consultation. In contrast doctors were more likely to have raised smoking for all age groups compared to younger smokers except the 80+ age group, where there was no evidence of any difference (Table 3).

For peer review only

However, for smokers over 70 years, doctors appeared not to offer any support to help quitting.

Despite discussing smoking with all age groups, doctors demonstrated marked differences in how they managed smokers (Figure 2). The probability of being prescribed NRT fell with increasing age (after 75 years) as did referral for counselling (after 70 years). Similar patterns were seen with prescription of NRT plus referral for counselling, but these estimates were imprecise due to small numbers. The odds ratio for not being advised to quit smoking was elevated after 65 years though for the 80+ age group: this was consistent with chance variation.

There was a marked gender difference - male respondents and their doctors were less likely to raise smoking during consultation and reported being given less support to quit smoking. A concordant pattern was also observed for social grade such that lower grade was associated with both patients and doctors raising smoking and modest evidence that they received more support than higher grades. Doctors appeared to be less likely to raise smoking with male patients and in more recent survey years.

In comparison to our original models, most of the results from the sensitivity analyses remained unchanged, the main difference being loss of statistical significance, due to reduced power.

Discussion

Our findings suggest that there are potential missed opportunities in facilitating smoking cessation in older smokers. We observed that older smokers, despite being less dependent on nicotine and smoking fewer cigarettes, key predictors of success in quitting[19], were also less likely to have attempted to quit. This was also partially reflected in less past experience with NRTs or referral for counselling. This may reflect reduced motivation as older smokers were less likely to raise smoking as a topic in their GP consultations but we did note that participants in the period around retirement were more likely to raise this. Transitional events such as retirement have been linked to success in quitting[20]. The relatively high success rate in quitting in this age group has been noted in the literature[20], but evidence is still limited[21]. Doctors appeared generally to be equitable in raising smoking as a topic across

all age-groups but were less likely to offer support for older participants. This was reflected in fewer prescriptions of NRTs and fewer referrals for counselling.

We cannot be certain whether these results demonstrate genuine inequities in receiving smoking cessation services for older smokers or reflect patient preferences which may be known to their doctor, hence no attempt to offer specific interventions. Previous studies have reported that medical professionals are less likely to advise older patients to stop smoking in a hospital setting[9] and less likely to prescribe NRT or other smoking cessation medications to those over 60 years[22,23], consistent with our observations.

Past research indicates that some older adults have low motivation to quit, believing either that 'the damage is done' and that quit attempts are unlikely to be successful after a lifetime of smoking, or that the harm of smoking is exaggerated or does not apply to them[19,22,23]. However, 'trigger events' such as an episode of ill health can still result in quit attempts, as can prompts from family and health professionals[9]. NRT has also been viewed with suspicion by some older smokers[9], but uptake can be high[24] and recent reviews of the evidence for effectiveness in older people showed that, although there is limited research in the oldest age groups, NRT appears to be effective in adults over 60 years[7,25].

We also noted important gender differences such that men were less likely to quit or cut down, less likely to use NRTs or any NHS cessation services, and less likely to speak to their GP about smoking. Some of this may reflect their greater level of nicotine dependency. This does not explain, however, why GPs were less likely to raise smoking with men and less likely to offer any form of support. More reassuringly, whilst participants of lower social grade were more nicotine dependent, they were more likely to speak to their GP about smoking and similarly GPs were more likely to raise this as a topic. We found no obvious social grade differences in GP referrals for counselling or prescription of NRTs. We had originally postulated "a priori" that it might be harder to get more recent cohorts to quit as the prevalent pool of smokers might be enriched with heavy smokers who find it difficult to quit. In fact, the time trends suggested the opposite pattern, as dependency is less in more recent cohorts.

Strengths and limitations

For peer review only

Because of the large sample size, we were able examine older age groups in far greater detail than in previous studies, allowing greater insights into patient and doctor behaviours. Previous studies on stopping smoking have typically aggregated all older age groups into one category of age 65 and older, even though this open-ended category is composed of many cohorts with different smoking initiation and cessation behaviours[7,12,26–28]. Secondly, we were able to estimate socioeconomic status using an occupation-based indicator, which previous research has found to be one of the strongest socioeconomic status measures - particularly for examining health issues in elderly populations in the UK[29]. Nevertheless, this measure captures only one dimension of socioeconomic status and a more multidimensional measure may have further reduced any residual confounding by socioeconomic status. The STS captures self-reported information on smoking behaviour and experience. These measures record patients' perceptions on interactions or lack thereof with their GP and are not based on direct observations or records of doctor-patient interactions. If older patients tend to recall or report their experiences differently from younger patients, the findings may be biased. For example, if older patients fail to recall doctor advice and hence this would exaggerate our observation that doctors provided less support for older participants. Secondly, our data are from an English population, so it is unclear whether our findings would be generalizable to the rest of the United Kingdom. The STS does not provide us data on co-morbidity or have any follow-up data on mortality. It is therefore possible that GPs choose not to try to change smoking behaviours in those patients who have multiple morbidities or a poor life expectancy, which may well be clinically appropriate. Finally, our analyses are based on respondents and we have no data on non-response bias. It is likely that this is greater for older respondents and those who did respond are probably healthier than the general older population. Whilst this may limit generalizability, it is likely that this sub-sample is of greater clinical interest as they are more likely to benefit from stopping smoking than frailer individuals with potentially more limited life expectancy.

Implications for research, policy and practice

It is unclear from our findings what proportion of smokers who were not offered interventions to stop smoking may have benefitted from them and would have wished to consider these options. Clearly smokers are under no obligation to try and quit but we suspect in some cases this may be due to not understanding the potential benefits or assuming there are none. Qualitative research could help to gain greater insight into why older patients and their GPs do not pursue smoking cessation, so that appropriate interventions can be designed

For peer review only

to reduce inequitable access. For example, recent research has demonstrated that the use of targeted invitations to stop smoking services increased attendance in all age groups, including people aged 65+, with the relative benefits larger in this age band than younger groups. This supports our view that it may be easier to change behaviour in this group if motivated.[30] Older adults need to be offered smoking cessation interventions that are acceptable, appropriate and available, even if they decide not to use them. With the re-commissioning of smoking cessation services, there is an opportunity to ensure that currently underserved groups are supported in attempts to stop smoking or reducing their intake. Evaluative studies are required to determine the most cost-effective type, location and mode of delivery of these interventions to ensure that older smokers can also realise the health benefits of quitting into their 60s and beyond.

Conclusions

This large national study has demonstrated that older smokers are less likely to quit or reduce consumption, though are less nicotine dependent. Potential opportunities to facilitate cessation are being missed as GPs are less likely to offer interventions or specific advice, though this may reflect past patient preferences. Service provision should consider how best to reduce these variations and a stronger effectiveness evidence base is required to support commissioning for this older population.

For peer review only

Acknowledgements

We thank Jamie Brown and Robert West of the Clinical, Educational & Health Psychology, Division of Psychology & Language Sciences, Faculty of Brain Sciences, University College London for their support with accessing the Smoking Toolkit Study. The Smoking Toolkit Study is currently primarily funded by Cancer Research UK (C1417/A14135; C36048/A11654; C44576/A19501), and has previously also been funded by Pfizer, GlaxoSmithKline, and the Department of Health. JB's post is funded by a fellowship from the Society for the Study of Addiction, and Cancer Research UK also provide support (C1417/A14135).

Competing interests

The authors declare they have no competing interests

Funding

This work was undertaken as part of the Ageing Well programme, funded by the NIHR School for Public Health Research (SPHR). The views expressed are those of the author(s) and not necessarily those of the NHS, the NIHR or the Department of Health.

Data sharing statement

The data that support these findings are available from the Smoking Toolkit Study. Restrictions apply to the availability of these data, which were used under licence for the current study, and so are not publicly available. Data are however available from the authors on reasonable request and with the permission of Robert West at robertwest100@googlemail.com

Contributor statement

The study was initially conceived and designed by YB-S, HJ and MH, with contributions from all authors. Data analysis was carried out by MH and YB-S.

All authors contributed to drafting and critically revising the paper.

All authors have approved the final version and are equally accountable for the work.

References

1. ASH. Smoking statistics. 2016.

For peer review only

2. Taylor DH, Hasselblad V, Henley SJ, et al. Benefits of smoking cessation for longevity. Am J Public Health. 2002;92(6):990–6.
3. Østbye T, Taylor DH. Smoking and Substance Abuse The Effect of Smoking on Years of Healthy Life (YHL) Lost among Middle-Aged and Older Americans. Health Serv Res. 2004;39(3):531–52.
4. US Department of Health and Human Services. The health benefits of smoking cessation. A report of the Surgeon General. 1990;627.
5. NICE. Stop smoking services. 2008.
6. Huddlestone L, Walker GM, Hussain-Mills R, Ratschen E. Treating tobacco dependence in older adults: a survey of primary care clinicians' knowledge, attitudes and practice. BMC Family Practice. 2015; 16:97 .
7. Cawkwell PB, Blaum C, Sherman SE. Pharmacological Smoking Cessation Therapies in Older Adults : A Review of the Evidence. Drugs Aging. Springer International Publishing; 2015;32(6):443–51. doi:10.1007/s40266-015-0274-9
8. ASH Scotland. Tobacco and Older Adults: a literature review. Edinburgh: ASH Scotland 2004.
9. Kerr S, Watson H, Tolson D, Lough M, Brown M. Smoking after the age of 65 years: a qualitative exploration of older current and former smokers' views on smoking, stopping smoking, and smoking cessation resources and services. Health Soc Care Community. 2006;14:572–82.
10. Twigg L, Moon G, Szatkowski L, Iggulden P. Smoking cessation in England: intentionality, anticipated ease of quitting and advice provision. Soc Sci Med. 2009; doi: 10.1016/j.socscimed.2008.11.032
11. Maguire CP, Ryan J, Kelly a, O'Neill D, Coakley D, Walsh JB. Do patient age and medical condition influence medical advice to stop smoking? Age Ageing 2000;29(3):264–6.
12. Huang Y, Britton J, Hubbard R, Lewis S. Who receives prescriptions for smoking cessation medications? An association rule mining analysis using a large primary care database. Tobacco Control 2013;22:274-279.
13. Wilson A, Hippisley-Cox J, Coupland C, Coleman T, Britton J, Barrett S. Smoking cessation treatment in primary care: prospective cohort study. Tobacco Control. 2005.14(4):242–6.
14. Gilbert H, Sutton S, Sutherland G. Who calls QUIT? The characteristics of smokers seeking advice via a telephone helpline compared with smokers attending a clinic and

For peer review only

those in the general population. Public Health. 2005;119(10):933–9.

15. Fidler J A, Shahab L, West O, Jarvis MJ, McEwen A, Stapleton J a, et al. "The smoking toolkit study": a national study of smoking and smoking cessation in England. BMC Public Health. 2011;11(1):479.

16. Heatherton TF, Kozlowski LT, Frecker RC, Rickert W, Robinson D. Measuring the Heaviness of Smoking: using self-reported time to the first cigarette of the day and number of cigarettes smoked per day. Addiction. 1989;84(7):791–800.

17. Heatherton TF, Kozlowski LT, Frecker RC, FargerstromKarl-Olov. The Fagerström test for nicotine dependence: a revision of the Fagerstrom Tolerance Questionnaire. Br J Addict. 1991;86(9):1119–27.

18. National Readership Survey. Social Grade. http://www.nrs.co.uk/nrs-print/lifestyle-and-classification-data/social-grade. Accessed 01 Oct 2016.

19. Hyland A, Borland R, Li Q, Yong H-H, McNeill A, Fong GT, et al. Individual-level predictors of cessation behaviours among participants in the International Tobacco Control (ITC) Four Country Survey. Tob Control. 2006;15 Suppl 3:iii83–94.

20. Lang I A, Rice NE, Wallace RB, Guralnik JM, Melzer D. Smoking cessation and transition into retirement: analyses from the English Longitudinal Study of Ageing. Age Ageing. 2007;36(6):638–43.

21. Zantinge E, Vn den Berg M, Smit A, Picavet HSJ. Retirement and a healthy lifestyle : opportunity or pitfall ? A narrative review of the literature. Eur J Public Heal. 2013;24(3):433–9.

22. Jarvis MJ, Wardle J, Waller J, Owen L. Prevalence of hardcore smoking in England, and associated attitudes and beliefs : cross sectional study. BMJ. 2003;326:1061.

23. Yong H, Borland R, Siahpush M. Quitting-related beliefs , intentions , and motivations of older smokers in four countries : findings from the international tobacco control policy evaluation survey. 2005;30:777–88.

24. Kotz D, Fidler J, West R. Factors associated with the use of aids to cessation in English smokers. Addiction. 2009;104(8):1403–10.

25. Zbikowski SM, Magnusson B, Pockey JR, Tindle HA, E WK. A review of smoking cessation interventions for smokers aged 50 and older. Maturitas. 2012;71:131–41.

26. Steinberg MB, Akincigil A, Delnevo CD, Crystal S, Carson JL. Gender and age disparities for smoking-cessation treatment. Am J Prev Med. 2006;30(5):405–12.

27. Chen D, Wu L-T. Smoking cessation interventions for adults aged 50 or older: a systematic review and meta analysis. Drug Alcohol Depend. 2015; 154:14-24.

For peer review only

28. Zhu S, Melcer T, Sun J, Rosbrook B, Pierce JP. Smoking Cessation With and Without Assistance A Population-Based Analysis. 2000;18(4):305–11.

29. Grundy E, Holt G. The socioeconomic status of older adults : How should we measure it in studies of health inequalities ? J Epidemiol Community Heal. 2001;55:895–904.

30. Gilbert H, Morris R et al. Effectiveness of personalised risk information and taster sessions to increase the uptake of smoking cessation services (Start2quit): a randomised controlled trial. The Lancet. 2017; 389:823-833.

For peer review only

Table 1: Association between socio-demographics and time period with smoking status and number of cigarettes consumed per day.

		Smoking Status								Cigarettes per day			
		Current vs. Never Smoker				Recent/Past vs. Never Smoker							
		OR	P-value	95% CI		OR	P-value	95% CI		OR*	P-value	95% CI	
Age													
	16-54	1.00				1.00				1.00			
	55-59	0.92	<0.001	0.88	0.97	1.79	<0.001	1.70	1.89	1.80	<0.001	1.68	1.93
	60-64	0.71	<0.001	0.68	0.75	2.19	<0.001	2.09	2.29	1.91	<0.001	1.79	2.04
	65-69	0.62	<0.001	0.59	0.66	2.65	<0.001	2.53	2.78	1.79	<0.001	1.66	1.92
	70-74	0.42	<0.001	0.39	0.44	2.41	<0.001	2.30	2.53	1.51	<0.001	1.39	1.64
	75-79	0.29	<0.001	0.27	0.31	2.35	<0.001	2.23	2.48	1.18	0.001	1.07	1.30
	80-84	0.21	<0.001	0.19	0.23	2.44	<0.001	2.29	2.60	0.94	0.330	0.82	1.07
	85+	0.13	<0.001	0.12	0.15	2.36	<0.001	2.19	2.54	0.73	0.001	0.61	0.89
	p-value for trend		<0.001				<0.001				<0.001		
Gender													
	Men	1.34	<0.001	1.31	1.37	1.39	<0.001	1.35	1.43	1.49	<0.000	1.44	1.54
	Women	1.00				1.00				1.00			
Social Grade													
	AB	1.00				1.00				1.00			
	C1	1.56	<0.001	1.50	1.63	1.00	0.897	0.97	1.04	1.03	0.416	0.96	1.09
	C2	2.40	<0.001	2.31	2.50	1.09	<0.001	1.05	1.14	1.31	<0.001	1.23	1.39
	D	2.88	<0.001	2.76	3.00	0.97	0.156	0.92	1.01	1.31	<0.001	1.23	1.40
	E	4.72	<0.001	4.52	4.92	0.96	0.078	0.92	1.00	1.62	<0.001	1.53	1.72
	p-value for trend		<0.001				0.008				<0.001		
Survey Years													
	2006-08	1.00				1.00				1.00			
	2009-10	0.88	<0.001	0.86	0.91	0.90	<0.001	0.86	0.94	0.86	<0.001	0.82	0.90
	2011-12	0.88	<0.001	0.86	0.91	1.14	<0.001	1.10	1.19	0.64	<0.001	0.61	0.68
	2013-15	0.80	<0.001	0.77	0.83	1.26	<0.001	1.22	1.31	0.55	<0.001	0.52	0.58
	p-value for trend		<0.001				<0.001				<0.001		

* Ordered logistic regression with following outcome categories: 10 or fewer cigarettes per day, 11-20 cigarettes per day, and 21 or more cigarettes per day.

For peer review only

Table 2: Association between socio-demographics and time period with nicotine dependency and quit attempts over the past year

		High nicotine dependency					Any quit attempts in past year			
		OR	P-value	95% CI			OR	P-value	95% CI	
Age										
	16-54	1.00					1.00			
	55-59	1.35	<0.001	1.25	1.44		0.74	<0.001	0.68	0.80
	60-64	1.31	<0.001	1.22	1.40		0.70	<0.001	0.64	0.76
	65-69	1.17	<0.001	1.08	1.26		0.65	<0.001	0.59	0.71
	70-74	0.98	0.669	0.90	1.07		0.57	<0.001	0.51	0.64
	75-79	0.69	<0.001	0.62	0.76		0.41	<0.001	0.35	0.48
	80-84	0.54	<0.001	0.47	0.63		0.27	<0.001	0.21	0.35
	85+	0.45	<0.001	0.37	0.56		0.32	<0.001	0.22	0.46
	p-value for trend		<0.001					<0.001		
Gender										
	Men	1.21	<0.001	1.17	1.26		0.81	<0.001	0.78	0.85
	Women	1.00					1.00			
Social Grade										
	ab	1.00					1.00			
	C1	1.20	<0.001	1.12	1.28		0.96	0.228	0.89	1.03
	C2	1.61	<0.001	1.51	1.72		0.91	0.014	0.85	0.98
	d	1.89	<0.001	1.77	2.02		0.88	0.001	0.81	0.95
	e	2.45	<0.001	2.29	2.61		0.90	0.005	0.84	0.97
	p-value for trend		<0.001					<0.001		
Survey Years										
	2006-08	1.00					1.00			
	2009-10	0.95	0.033	0.91	1.00		0.80	<0.001	0.76	0.84
	2011-12	0.80	<0.001	0.76	0.85		0.73	<0.001	0.69	0.77
	2013-15	0.72	<0.001	0.68	0.76		0.85	<0.001	0.81	0.90
	p-value for trend		<0.001					<0.001		

For peer review only

Table 3: Association between socio-demographics and time period with discussion of smoking at GP consultation and whether the doctor offered any active management.

		Patient raised smoking				Doctor raised smoking				Doctor no support			
		OR	P-value	95% CI		OR	P-value	95% CI		OR	P-value	95% CI	
Age													
	16-54	1.00				1.00				1.00			
	55-59	1.31	0.006	1.08	1.58	1.58	<0.001	1.44	1.74	1.14	0.126	0.96	1.34
	60-64	1.47	<0.001	1.22	1.78	1.88	<0.001	1.72	2.07	1.04	0.636	0.89	1.22
	65-69	1.05	0.666	0.84	1.31	1.98	<0.001	1.78	2.19	0.90	0.233	0.75	1.07
	70-74	0.96	0.774	0.73	1.27	1.85	<0.001	1.63	2.09	1.27	0.023	1.03	1.55
	75-79	0.49	0.002	0.31	0.78	1.69	<0.001	1.44	1.98	1.87	<0.001	1.43	2.44
	80+	0.48	0.005	0.28	0.80	1.02	0.804	0.85	1.24	3.16	<0.001	2.20	4.55
	p-value for trend		<0.001				<0.001				<0.001		
Gender													
	Men	0.81	<0.001	0.74	0.90	1.72	<0.001	0.69	0.76	1.15	0.002	1.05	1.25
	Women	1.00				1.00				1.00			
Social Grade													
	ab	1.00				1.00				1.00			
	C1	1.12	0.259	0.92	1.36	1.02	0.724	0.93	1.11	0.96	0.614	0.81	1.13
	C2	1.01	0.897	0.83	1.24	1.06	0.190	0.97	1.16	0.87	0.097	0.73	1.03
	d	1.24	0.036	1.01	1.51	1.09	0.073	0.99	1.19	0.90	0.210	0.75	1.06
	e	1.70	<0.001	1.40	2.07	1.47	<0.001	1.35	1.60	0.83	0.023	0.70	0.97
	p-value for trend		<0.001				<0.001				0.017		
Survey Years													
	2009-10					1.00				1.00			
	2011-12					0.88	<0.001	0.83	0.93	0.99	0.925	0.89	1.12
	2013-15					0.79	<0.001	0.75	0.84	1.18	0.007	1.05	1.32
	p-value for trend						<0.001				<0.001		
	2012-13	1.00											
	2014-15	0.85	0.003	0.77	0.95								

For peer review only

Supplemental Tables

Supplemental Table 1: Descriptive statistics of sociodemographics and time period with smoking status, past quitting and use of cessation therapies or GP management

		Ever Smoker	Nicotine Dependency	Quit attempts in past year	Past NRT use	Past NHS cessation clinics use	Respondent raised the topic of smoking with GP	GP spoke to respondent about quitting smoking	GP referrals to smoking cessation therapies
		N=68,967	N=51,920	N=43,385	N=20,286	N=16,026	N=11,432	N=29,578	N=10,466
**Age**		%	%	%	%	%	%	%	%
	**16-54**	61.4	69.4	74.6	75.8	80.1	73.6	73.7	69.2
	**55-59**	7.0	6.8	6.6	6.9	5.8	6.7	6.8	7.5
	**60-64**	8.2	7.2	6.4	6.4	5.4	6.4	6.6	7.6
	**65-69**	7.7	6.1	5.1	5.0	4.1	5.8	5.4	6.6
	**70-74**	6.2	4.5	3.6	3.1	2.6	3.6	3.8	4.6
	**75-79**	4.7	3.2	2.2	1.7	1.2	2.1	2.2	2.7
	**80-84**	3.0	1.8	1.1	0.7	0.4	1.0	1.1	1.2
	**85+**	1.9	0.9	0.5	0.3	0.2	0.7	0.6	0.7
	**Total**	100.0	100.0	100.0	100.0	100.0	100.0	100.0	100.0
**Gender**		%	%	%	%	%	%	%	%
	**Men**	48.0	51.0	50.5	48.4	47.2	54.1	52.0	47.4
	**Women**	52.0	49.0	49.5	51.6	52.8	46.0	48.0	52.7
	**Total**	100.0	100.0	100.0	100.0	100.0	100.0	100.0	100.0
**Social Grade**		%	%	%	%	%	%	%	%
	**AB**	15.2	12.4	10.5	10.6	10.9	10.8	10.6	10.5
	**C1**	24.9	23.8	22.9	22.9	23.7	24.8	22.8	21.8
	**C2**	22.2	22.5	22.7	22.6	22.7	23.7	22.4	21.5
	**D**	16.9	18.3	19.3	18.8	18.9	20.0	19.4	18.9
	**E**	20.8	23.1	24.6	25.1	23.8	20.7	24.8	27.3
	**Total**	100.0	100.0	100.0	100.0	100.0	100.0	100.0	100.0
**Survey Year**		%	%	%	%	%	%	%	%
	**2006**	2.1	2.8	2.3	2.6	2.8			
	**2007**	13.3	17.6	14.4	16.2	16.5			
	**2008**	9.9	13.1	11.1	12.4	11.8			
	**2009**	10.5	13.9	11.9	13.1	11.8		11.7	
	**2010**	13.7	13.2	14.0	14.6	13.4		20.2	21.9
	**2011**	12.5	10.2	12.2	12.6	10.8		17.1	19.0
	**2012**	11.9	9.9	11.9	8.1	10.8	12.0	17.0	20.0
	**2013**	12.7	9.3	10.7	9.2	11.0	42.2	16.3	18.4
	**2014**	10.8	8.2	9.4	9.2	9.4	37.1	14.3	16.7
	**2015**	2.8	1.9	2.2	2.0	1.8	8.7	3.3	4.1
	**Total**	100.0	100.0	100.0	100.0	100.0	100.0	100.0	100.0

For peer review only

Supplemental Table 2: Odds ratios for sociodemographics and time period with past use of NRTs or stop smoking counselling services

		Past NRT use					Past NHS advice services use			
		**OR**	**P-value**	**95% CI**			**OR**	**P-value**	**95% CI**	
**Age**										
	**16-54**	1					1			
	**55-64**	1.132	0.012	1.028	1.247		1.555	<0.001	1.303	1.856
	**65-74**	1.086	0.182	0.962	1.227		1.378	0.007	1.089	1.742
	**75+**	0.703	0.003	0.559	0.886		0.994	0.981	0.613	1.613
**Gender**										
	**Men**	0.903	0.003	0.845	0.966		0.773	<0.001	0.680	0.879
	**Women**	1					1			
**Social Grade**										
	**AB**	1					1			
	**C1**	0.884	0.047	0.783	0.999		0.920	0.478	0.731	1.158
	**C2**	0.887	0.054	0.785	1.002		0.890	0.327	0.706	1.123
	**D**	0.801	0.001	0.705	0.909		1.059	0.630	0.838	1.338
	**E**	0.899	0.081	0.797	1.013		0.973	0.815	0.776	1.221
**Survey Years**										
	**2006-08**	1					1			
	**2009-10**	1.065	0.138	0.980	1.158		0.885	0.122	0.758	1.033
	**2011-12**	0.839	<0.001	0.764	0.922		0.612	<0.001	0.512	0.733
	**2013-15**	0.597	<0.001	0.540	0.661		0.495	<0.001	0.409	0.599

For peer review only

Supplemental Table 3: Odds ratios for sociodemographics with different GP management compared to no referral

		GP referrals to help quit smoking															
		Counselling vs. No Referral				Pharmacology vs. No Referral				Counselling and Pharmacology vs. No Referral				GP did not advise to quit smoking vs. No Referral			
		OR	P-value	95% CI		OR	P-value	95% CI		OR	P-value	95% CI		OR	P-value	95% CI	
Age																	
	16-54	1				1				1				1			
	55-59	0.869	0.126	0.726	1.040	0.814	0.068	0.653	1.016	1.331	0.125	0.924	1.916	1.102	0.428	0.866	1.403
	60-64	0.944	0.515	0.794	1.123	0.864	0.179	0.697	1.069	1.821	<0.001	1.318	2.515	1.097	0.443	0.866	1.389
	65-69	1.034	0.734	0.851	1.258	1.077	0.530	0.855	1.356	2.127	<0.001	1.497	3.024	1.391	0.010	1.081	1.789
	70-74	0.690	0.002	0.547	0.869	0.854	0.244	0.654	1.114	1.390	0.137	0.900	2.146	1.476	0.004	1.128	1.930
	75-79	0.512	<0.001	0.377	0.696	0.551	0.002	0.380	0.799	0.618	0.201	0.296	1.291	1.636	0.002	1.190	2.248
	80+	0.361	<0.001	0.241	0.539	0.251	<0.001	0.138	0.456	0.109	0.028	0.015	0.787	1.309	0.191	0.875	1.959
Gender																	
	Men	0.820	<0.001	0.744	0.904	0.991	0.886	0.882	1.115	0.798	0.034	0.647	0.983	0.960	0.547	0.842	1.095
	Women	1				1				1				1			
Social Grade																	
	AB	1				1				1				1			
	C1	1.064	0.510	0.884	1.280	1.019	0.874	0.810	1.282	0.995	0.979	0.691	1.434	0.946	0.651	0.744	1.203
	C2	1.182	0.077	0.982	1.422	1.225	0.080	0.976	1.536	0.697	0.068	0.473	1.027	0.894	0.366	0.701	1.140
	D	1.129	0.208	0.935	1.363	1.184	0.153	0.939	1.491	0.728	0.112	0.492	1.077	0.908	0.444	0.708	1.163
	E	1.214	0.033	1.016	1.451	1.311	0.015	1.054	1.631	0.860	0.404	0.603	1.226	0.978	0.849	0.776	1.232
Survey Years																	
	2009-10	1				1				1				1			
	2011-12	0.899	0.101	0.792	1.021	1.325	<0.001	1.137	1.543	0.808	0.087	0.633	1.031	0.847	0.060	0.712	1.007
	2013-15	0.899	0.103	0.791	1.022	0.880	0.120	0.750	1.034	0.408	<0.001	0.307	0.540	0.983	0.844	0.829	1.166

For peer review only

Box 1 Definitions of terms and outcomes

Nicotine dependency (Yes/No) Derived from Heaviness of Smoking Index (HSI) combining two scales (0-3) on "How soon after you wake up do you smoke your first cigarette?" (sooner gets higher score) and "How many cigarettes per day do you usually smoke?" (more cigarettes gets higher score). Scores from both questions are added for each respondent (min=0, max=6) and we dichotomized this variable into a high (3-6) and low (0-2) score.

Quit attempt (Yes/No) This was derived from the following question, "How many serious attempts to stop smoking have you made in the last 12 months?" asked to current smokers and recent smokers (in last 12 months). This was dichotomised as any serious attempts to stop smoking within the last year or no attempts.

Cutting down (Yes/No). This was only asked of current smokers and was coded from the question "Are you trying to cut down on how much you smoke?" (waves 1-32) which was later changed to "Are you currently trying to cut down on how much you smoke but not currently trying to stop?" (waves 33-66, 68, 70, 72, 74, 76, 78-102)

Use of NRTs (Yes/No) Respondents who were current smokers trying to cut down could report use of one or more of the following NRT products: Nicotine replacement gum, lozenges/tablets, inhaler, nasal spray, patch, or mouth spray. We dichotomised use of any NRT products versus none (question not included in waves 67, 69, 71, 73, 75, and 77).

Use of NHS advice services (Yes/No) Current smokers who responded that they had at least one serious quit attempt were shown a list of potential cessation services: NHS Stop Smoking Service group; one to one counselling/advice support session; contact with local NHS Stop Smoking Services; NHS smoking helpline; or Quitline. Use of NHS Smoke Free website was not included in this list because it was available to a small subset of the survey (waves 26-102).

Patient raised smoking (Yes/No) This was captured by a dichotomous variable to the question "Have you raised the topic of smoking with your GP in the past year (i.e. last 12 months)?" asked of current smokers and recent ex-smokers (quit within last 12 months).

Doctor raised smoking (Yes/No) This came from the question, "Has your GP spoken to you about smoking in the past year (i.e. last 12 months)?" This question was asked in waves 31-39 to current and former smokers (quit within last 12 months or more) and in waves 40-102 to current smokers and recent ex-smokers (quit within last 12 months).

Doctor management If Doctor raised smoking was YES then the following responses were available: (i) he/she suggested that I go to a specialist stop smoking advisor or group or that I see a nurse in the practice (**Counselling**); (ii) he/she offered me a prescription for a nicotine patch, nicotine gum, or other nicotine product (**NRT Prescription**); (iii) he/she suggested that I go to a specialist stop smoking advisor or group, or see a nurse in the practice and offered me a prescription for a nicotine patch, nicotine gum or another nicotine product (**Counselling and NRT prescription**); (iv) Yes, he/she advised me to stop but did not offer anything (**Advice only**); (v) Yes, he/she asked me about my smoking but did not advise me to stop smoking (**no active management**). These responses are available in waves 40-102 to current smokers and recent ex-smokers (quit within last 12 months).

Legend for Figures

Figure 1: Odds ratios for past use of nicotine replacement therapies (NRTs) and NHS stop smoking clinics by age group

Figure 2: Odds ratios for GP management (NRTs, counselling, both or no advice to quit) of smokers by age groups.

For peer review only

For peer review only

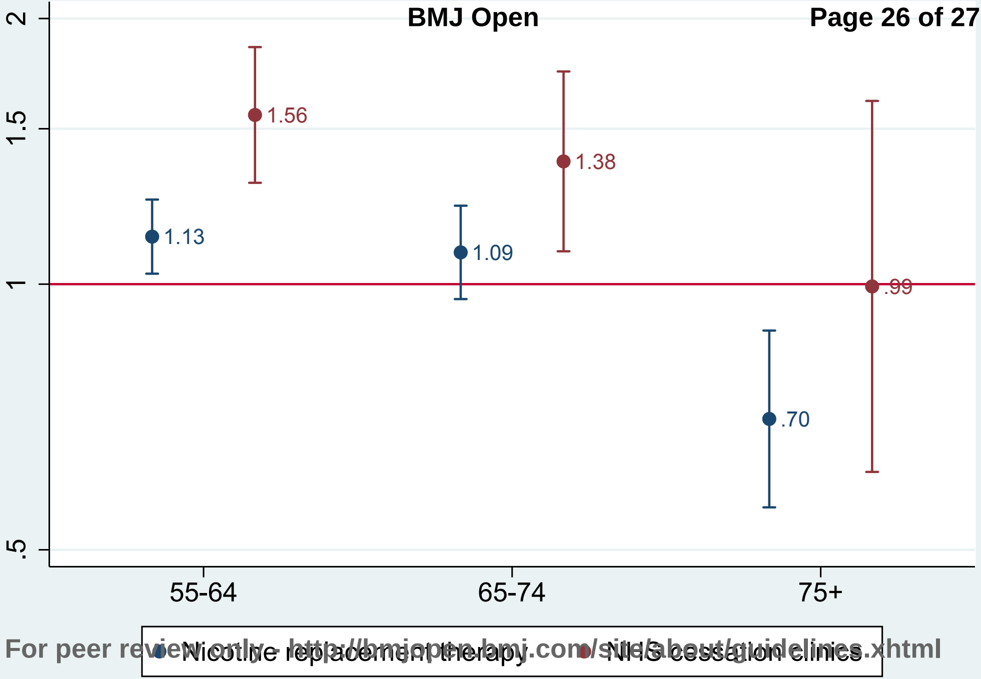

Notes: Age reference is 16-54 years
Models are adjusted for gender, social grade, and survey year

For peer review only - http://bmjopen.bmj.com/site/about/guidelines.xhtml

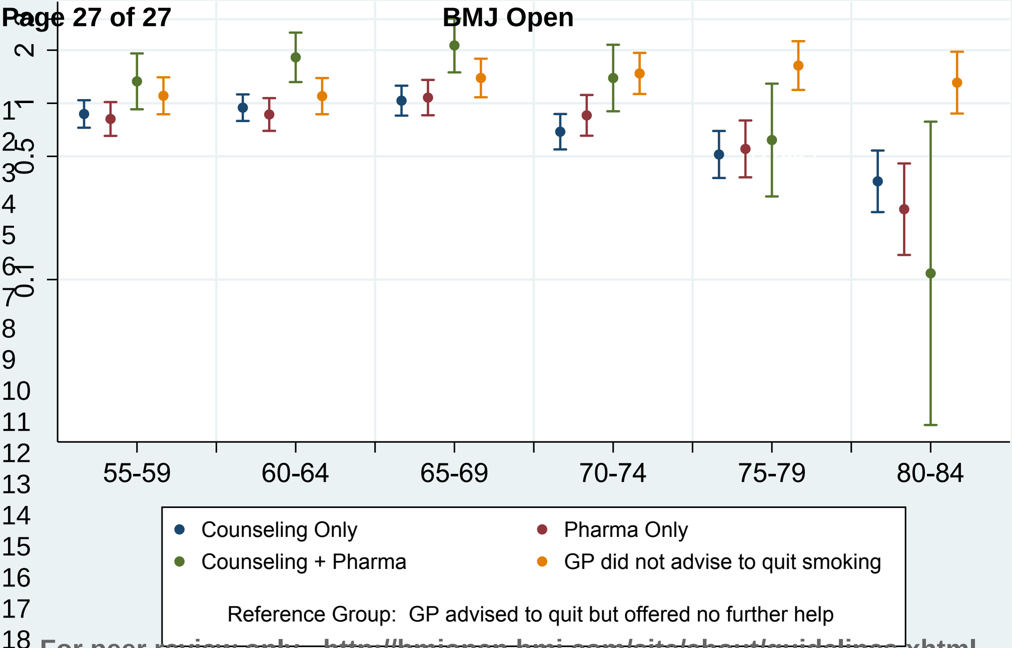

For peer review only - http://bmjopen.bmj.com/site/about/guidelines.xhtml

Notes: Age reference is 16-54 years
Models are adjusted for gender, social grade, and survey year

BMJ Open

What are older smokers' attitudes to quitting and how are they managed in primary care? An analysis of the cross-sectional English Smoking Toolkit Study

Journal:	*BMJ Open*
Manuscript ID	bmjopen-2017-018150.R1
Article Type:	Research
Date Submitted by the Author:	07-Sep-2017

Complete List of Authors:	Jordan, Hannah; University of Sheffield, School of Health and Related Research<br>Hidajat, Mira; University of Bristol, School of Social and Community Medicine<br>Payne, Nick; University of Sheffield, School of Health and Related Research (ScHARR)<br>Adams, J; University of Cambridge, Centre for Diet & Activity Research<br>White, Martin; University of Cambridge School of Clinical Medicine, MRC Epidemiology Unit<br>Ben-Shlomo, Yoav; University of Bristol,

**Primary Subject Heading**:	Smoking and tobacco
Secondary Subject Heading:	Public health, General practice / Family practice, Health services research
Keywords:	PUBLIC HEALTH, Smoking cessation, Inequalities, Aging, PRIMARY CARE

SCHOLARONE™
Manuscripts

What are older smokers' attitudes to quitting and how are they are managed in primary care? An analysis of the cross-sectional English Smoking Toolkit Study

Hannah Jordan[1], Mira Hidajat[2], Nick Payne[1], Jean Adams[3], Martin White[3], Yoav Ben-Shlomo[2]

Affiliations

1. Section of Public Health, School of Health and Related Research (ScHARR), The University of Sheffield, Regent Court, 30 Regent Street, Sheffield S1 4DA
2. School of Social and Community Medicine, University of Bristol, 39 Whatley Road, Bristol BS8 2PS,
3. MRC Epidemiology Unit, University of Cambridge School of Clinical Medicine, Box 285 Institute of Metabolic Science, Cambridge Biomedical Campus, Cambridge, CB2 0QQ

HJ is the corresponding author (email: h.c.jordan@sheffield.ac.uk)

For peer review only

Abstract

Objectives

To investigate whether age is associated with access to smoking cessation services.

Design

Data from the Smoking Toolkit Study 2006-15, a repeated multi-wave cross-sectional household survey (N=181,157).

Setting

England

Participants

Past-year smokers who participated in any of the 102 waves stratified into age groups.

Outcome measures

Amount smoked and nicotine dependency, self-reported quit attempts and use of smoking cessation interventions. Self-report of whether the GP raised the topic of smoking and made referrals for pharmacological support (prescription of NRTs) or other support (counselling or support groups).

Results

Older smokers (75 + years) were less likely to report that they were attempting to quit smoking or seek help from a GP, despite being less nicotine dependent. GPs raised smoking as a topic equally across all age groups, but smokers aged 70 plus were more likely not to be referred for NRT or other support (odds ratios relative to 16-54 years; 70-74 years 1.27, 95%CI 1.03, 1.55; 75-79 1.87, 95% CI 1.43, 2.44; 80+ 3.16, 95% CI 2.20, 4.55; p-value for trend <0.001).

Conclusions

Our findings suggest that there are potential missed opportunities in facilitating smoking cessation in older smokers. In this large population based study older smokers appeared less interested in quitting and were less likely to be offered support, despite being less addicted to nicotine than younger smokers. It is unclear whether this constitutes inequitable access to

For peer review only - http://bmjopen.bmj.com/site/about/guidelines.xhtml
For peer review only

services or reflects informed choices by older smokers and their general practitioners. Future research is needed to understand why older smokers and GPs do not pursue smoking cessation. Service provision should consider how best to reduce these variations and a stronger effectiveness evidence base is required to support commissioning for this older population so that, where appropriate, older smokers are not missing out on smoking cessation therapies and the health benefits of cessation at older ages.

Strengths and limitations of this study

- The findings of the study are based on a large, representative survey of the adult population of England
- The large sample size enabled us to examine older age groups in far greater detail than in previous studies
- Quit behaviours and support were self-reported, raising questions of differences in recall or reporting behaviour among older smokers

For peer review only - http://bmjopen.bmj.com/site/about/guidelines.xhtml

Introduction

Despite a marked decline in smoking behaviour over the last few decades, around 10 million adults in the UK still smoke, of which 11% (1.1 million) are over the age of 60[1]. There is, however, clear evidence for the benefits of quitting smoking at older ages. Large scale prospective cohort studies have found that smokers who quit after the age of 65 years benefit from reduced mortality (2-4 extra years of life)[2] additional healthy life years[3] and reduced morbidity[4]. Although the relative risks associated with smoking status decline with age, the absolute risk differences continue to increase[4].

The National Institute for Health and Care Excellence (NICE) recommends that smoking cessation advice should be provided to all smokers, regardless of age. However current policy aims to target specific groups, such as pregnant women and socio-economically disadvantaged populations for cessation advice whilst older smokers are not generally recognised as a priority group[5]. A recent study reported that opportunities to offer cessation advice and support to older smokers may often be missed in Primary Care [6]. However, a recent review showed that pharmacotherapy such as Nicotine Replacement Therapy (NRT) was effective in older adults, but that the literature was sparse and effective smoking cessation services may need to be tailored to the needs of older adults[7].

There are several possible reasons why smoking cessation therapies may not be used equally across age groups. It has been suggested that older smokers may be more strongly addicted to nicotine, which may hamper attempts to initiate quitting[8]. Older people themselves may have beliefs about quitting, considering themselves ‘survivors’, or believing that ‘the damage is done’ so that they see no point in attempting to quit later in life, resulting in reluctance to demand services or recognise the benefits of quitting[9]. Health professionals may have beliefs about older smokers that hinder their access to smoking cessation services, such as a reluctance to give cessation advice or to provide medication[10–13]. The type, location and visibility of smoking cessation services is decided locally [14], and may introduce barriers by age. For example, older smokers may be reluctant to use telephone or online support such as text messaging[15].

For peer review only - http://bmjopen.bmj.com/site/about/guidelines.xhtml

For peer review only

In this paper, we examine the potential reasons why older smokers may or may not have equitable access to smoking cessation therapies in a large, multi-wave, national cross-sectional survey in England. Because of the large sample size, we were able examine older age groups in far greater detail than in previous studies, using five year age bands up to age 80+ rather than aggregating data for those over 65 or even younger. We compare older smokers to younger smokers to test if they are as likely to: (a) attempt to quit or cut down on their cigarette consumption? (b) be nicotine dependent? (c) seek help from a general practitioner? In addition, we test whether general practitioners are as likely, when comparing older to younger smokers to: (d) discuss smoking cessation; and (e) refer patients to either smoking cessation services and/or prescribe NRTs?

Methods

This manuscript was written in adherence to the STROBE (Strengthening the Reporting of Observational Studies in Epidemiology) statement [16].

Data

We used data from the Smoking Toolkit Study (STS), a repeated, multi-wave, cross-sectional household survey conducted approximately every month from November 2006 to January 2015 across England (102 waves of data, N=181,157)[15]. Each survey wave contains responses from approximately 1,800 adults aged 16+ (approximately 570 ex-smokers and 500 current smokers) collected during a face-to-face computer-assisted household survey. The sampling method is a hybrid between random probability and simple quota. The sample is identified by a random selection of over 170,000 localities (‘output areas’) after stratification by a geo-demographic analysis of the population. Each locality contains approximately 250 households. Interviewers visit households within the locality starting at a random point in the area. One member per household is interviewed until interviewers achieve local quotas designed to minimise differences in the probability of participation. The method has been shown to result in a sample that is nationally representative in its socio-demographic composition. Ethical approval was granted to the STS, and the anonymised study data were used under licence. Although the data are not publically available, they can be requested from the STS [17]

Exposure

For peer review only - http://bmjopen.bmj.com/site/about/guidelines.xhtml

Our exposure of interest was age. We chose to operationalise age into bands, not assuming any linear relationship, as follows: 16 to 54 years as baseline then 5 year bands from 55 to 79, and 80+ as the oldest age group. Age bands were selected to allow us to look in detail at older smokers' experience, while retaining sufficient numbers in each band for analysis.

Smoking status, nicotine dependency and reducing consumption

We classified smoking status using the four categories in the STS: current smoker (respondents who indicated that they smoke cigarettes (including hand-rolled) or other forms of tobacco (e.g. pipe or cigar); recent smoker (respondents who indicated that they have stopped smoking within the past year); past smoker (respondents who indicated that they have not smoked for a year or more) and never smokers. For current, recent and past smokers, we categorized respondents into groups by the amount smoked: 10 or fewer cigarettes per day, 11-20 cigarettes per day, and 21 or more cigarettes per day.

Dependency and past use of services

Nicotine dependency (Yes/No) was classified using the Heaviness of Smoking Index (HSI)[18] , a validated index based on a subset of questions from the Fagerstrom Test of Nicotine Dependency[19]. (See box 1 for more details concerning specific outcomes).

We defined smoking cessation and reduction behaviours - quit attempts and cutting down in the past 12 months - as dichotomous variables due to small numbers of respondents with more than one quit attempt.We classified whether current smokers used two types of smoking cessation interventions (Yes/No) - use of NRTs and NHS stop smoking counselling.

GP – patient interaction and management

The survey captures both patient and GP initiated interactions around smoking cessation, allowing us to examine whether relevant respondents raised the topic of smoking with their GP and also whether the doctor raised the issue of smoking. For the latter question there were a series of outcome options such as a referral for stop smoking counselling, prescription of a NRT, both, or advice but no referral. To look at the determinants of advice only, we created a dichotomous variable by comparing no referral for counselling, prescription of NRT or both. Finally there was an option that the GP did not specifically advise the patient to stop smoking (no active management).

For peer review only - http://bmjopen.bmj.com/site/about/guidelines.xhtml

Potential confounders

We examined the following potential confounders: gender, secular period as measured by year of survey (two year bands) and a measure of socioeconomic status ('social grade' – see below). We argued that both social grade and gender could have influenced past smoking behaviour and may determine willingness to engage in cessation therapies as well as being age-related (more women and more affluent older participants). Secular period may influence availability of cessation therapies such as counselling as services are re-procured and reconfigured over time.

Social grade was reported using the five occupation-based categories of the National Readership Survey method: AB (higher/intermediate managerial, administrative or professional), C1 (supervisory, clerical and junior managerial, administrative and professional), C2 (skilled manual workers), D (Semi-skilled and unskilled manual workers), and E (state pensioners, casual and lowest grade workers, unemployed with state benefits only[20]). The social grade of the household head was used to classify the entire household.

Statistical analyses

We conducted logistic regression analyses based on the relevant outcome variables (nicotine dependency, quit attempts, cutting down, past NRT usage, past NHS advice services used, patient or GP raised smoking, GP prescription of NRT, stop smoking counselling or just advice). Independent variables were age, gender, social grade and survey year. We modelled age group both as a dummy variable and as an ordinal variable to test for a linear trend. Ordered logistic regression was performed for categories of amount smoked per day. To examine how management differed by age group we derived the odds ratios (ORs) (95% CI, p-value), using logistic regression models, for referral for counselling, NRT prescription, both or no active management, as compared to the baseline group of the GP giving advice to quit (advice only) but no referral. For each model we ran unadjusted and adjusted models conditioning on gender, social grade and secular period. Due to questionnaire changes, some questions were not available in all waves, particularly doctor-patient interactions, NHS stop smoking services use, and NRT use. In our analyses, we used the appropriate waves for each model accordingly although it meant using different waves of data across the various models (see Box 1). We conducted a sensitivity analysis using only the survey waves with complete data for GP-related questions (waves 72-102).

For peer review only - http://bmjopen.bmj.com/site/about/guidelines.xhtml

For peer review only

Results

Descriptive analyses

From a total sample of 181,157 participants, there were 41,031 (22.7%) current smokers, 2,825 (1.6%) recent smokers, 25,367 (14.0%) past smokers and 112,046 (61.9%) never smokers and 131 missing (0.07%). Older participants were less likely to be current smokers, more likely to be former smokers and there was an inverted J shaped relationship for smoking intensity, with 55-59 year olds and those >65 years old less likely to be heavy smokers (Table 1). Supplemental Table 1 describes each outcome by socio-demographic characteristics and survey year. Numbers of participants included in each analysis depend on numbers of respondents for individual survey questions.

Nicotine dependency and quit attempts

Nicotine dependency data were available for 51,920 people. Dependency showed an inverted J pattern so that participants aged 55 to 69 appeared to have higher nicotine dependency but from 70 years upwards there was a progressive reduction in the odds ratio for dependency (Table 2). However, older participants from 55 years onwards were less likely to report having at least one quit attempt (Compared to 16-54 year olds, the OR for quit attempts in the past year for 55-59 year olds 0.74 (0.68-0.80), reducing to 0.32 (0.22-0.46) for those age 85+). Men showed greater dependency and were less likely to quit. The odds of high nicotine dependency increased from higher to lower social grades and respondents from the lower social grades were also less likely to report a recent quit attempt. Participants from surveys in more recent years were less dependent and less likely to make quit attempts.

Past use of NRT or stop smoking counselling

Past use of NRTs was calculated on data from 20,286 participants. Use was more common among 55 to 64 year olds compared to 16 to 54 year olds (OR 1.13 (1.03-1.25)); use of stop smoking counselling (16,026 participants) was more common among 65 to 74 year olds (OR 1.38 (1.09-1.74)). Older participants (75 + years) were less likely to have been prescribed NRT than 55-64 or 65-74 year olds (Figure 1, OR 0.70). Data on participants over the age of 75 was combined due to small numbers in the oldest age groups. We found similar patterns by gender; men were less likely than women to have been prescribed NRTs or referred for counselling (Supplemental Table 2). Participants in social grade D were less likely to have been prescribed NRTs than those in social grade AB but there were no social grade differences in referral for counselling. There appeared to be a secular pattern so that

For peer review only - http://bmjopen.bmj.com/site/about/guidelines.xhtml

respondents from recent survey years (2011-15) were less likely to have been prescribed NRTs and referred for counselling than those from 2006-08.

Raising smoking in consultations and management

We found an inverted J-shaped relationship for age group, such that older adults (75 years and above) were much less likely to have raised the topic of smoking during their GP consultation (OR for 75-79 years compared to 16-54 years 0.49 (0.31-0.78). In contrast GPs were more likely to have raised smoking for all age groups compared to younger smokers except the 80+ age group, where there was no evidence of any difference (Table 3).

However, smokers over 70 years were less likely to report that their GPs offered any support to help quitting.

Despite discussing smoking with all age groups, GPs demonstrated marked differences in how they managed smokers. The self-reported probability of being prescribed NRT fell with increasing age (after 75 years) as did referral for counselling (after 70 years). Similar patterns were seen with prescription of NRT plus referral for counselling, but these estimates were imprecise due to small numbers. The odds ratio for not being advised to quit smoking was elevated after 65 years though for the 80+ age group: this was consistent with chance variation (Figure 2; Supplemental table 3).

There was a marked gender difference – reports from male respondents indicate that they and their GPs were less likely to raise smoking during consultation. Men also reported being given less support to quit smoking. A concordant pattern was also observed for social grade such that lower grade was associated with both patients and doctors raising smoking and modest evidence that they received more support than higher grades. GPs appeared to be less likely to raise smoking with male patients and in more recent survey years.

In comparison to our original models, most of the results from the sensitivity analyses remained unchanged, the main difference being loss of statistical significance, due to reduced power (Supplemental table 4).

Discussion

For peer review only - http://bmjopen.bmj.com/site/about/guidelines.xhtml

Our findings suggest that there are potential missed opportunities in facilitating smoking cessation in older smokers. We observed that older smokers, despite being less dependent on nicotine and smoking fewer cigarettes, key predictors of success in quitting[21], were also less likely to have attempted to quit. This was also partially reflected in less past experience with NRTs or referral for counselling. This may reflect reduced motivation as older smokers were less likely to raise smoking as a topic in their GP consultations but we did note that participants in the period around retirement were more likely to raise this. Transitional events such as retirement have been linked to success in quitting[22]. The relatively high success rate in quitting in this age group has been noted in the literature[20], but evidence is still limited[23]. GPs appeared generally to be equitable in raising smoking as a topic across all age groups but older participants were less likely to report being supported to quit. This was reflected in fewer prescriptions of NRTs and fewer referrals for counselling.

We cannot be certain whether these results demonstrate genuine inequities in receiving smoking cessation services for older smokers or reflect patient preferences which may be known to their GP, hence no attempt to offer specific interventions. Previous studies have reported that medical professionals are less likely to advise older patients to stop smoking in a hospital setting[9] and less likely to prescribe NRT or other smoking cessation medications to those over 60 years[24,25], consistent with our observations.

Past research indicates that some older adults have low motivation to quit, believing either that 'the damage is done' and that quit attempts are unlikely to be successful after a lifetime of smoking, or that the harm of smoking is exaggerated or does not apply to them[21,24,25]. However, 'trigger events' such as an episode of ill health can still result in quit attempts, as can prompts from family and health professionals[9]. NRT has also been viewed with suspicion by some older smokers[9], but uptake can be high[26] and recent reviews of the evidence for effectiveness in older people showed that, although there is limited research in the oldest age groups, NRT appears to be effective in adults over 60 years[7,27]. Although the benefits of smoking cessation for life expectancy accumulate with years since quitting, and there is still relatively little evidence on the benefits to people over the age of 65, it has been argued that smoking related risks such as myocardial infarction and vascular disease are not modified by age [28] and that healthy life years may be important to emphasise for an older population[3].

For peer review only - http://bmjopen.bmj.com/site/about/guidelines.xhtml

We also noted important gender differences such that men were less likely to quit or cut down, less likely to use NRTs or any NHS cessation services, and less likely to speak to their GP about smoking. Some of this may reflect their greater level of nicotine dependency. This does not explain, however, why GPs were reportedly less likely to raise smoking with men and less likely to offer any form of support. More reassuringly, whilst participants of lower social grade were more nicotine dependent, they were more likely to speak to their GP about smoking and similarly GPs were more likely to raise this as a topic. We found no obvious social grade differences in GP referrals for counselling or prescription of NRTs. We had originally postulated "a priori" that it might be harder to get more recent cohorts to quit as the prevalent pool of smokers might be enriched with heavy smokers who find it difficult to quit. In fact, the time trends suggested the opposite pattern, as dependency is less in more recent cohorts.

Strengths and limitations

Because of the large sample size, we were able examine older age groups in far greater detail than in previous studies, allowing greater insights into patient and GP behaviours. Previous studies on stopping smoking have typically aggregated all older age groups into one category of age 65 and older, even though this open-ended category is composed of many cohorts with different smoking initiation and cessation behaviours[7,12,29–31].

Secondly, we were able to estimate socioeconomic status using an occupation-based indicator, which previous research has found to be one of the strongest socioeconomic status measures - particularly for examining health issues in elderly populations in the UK[32]. Nevertheless, this measure captures only one dimension of socioeconomic status. A more multidimensional measure may have further reduced any residual confounding by socioeconomic status, but without postcode data we were not able to add this.

The STS captures self-reported information on smoking behaviour and experience. These measures record patients' perceptions on interactions or lack thereof with their GP and are not based on direct observations or records of doctor-patient interactions. If older patients tend to recall or report their experiences differently from younger patients, the findings may be biased. For example, if older patients fail to recall GP advice and hence this would exaggerate our observation that GPs provided less support for older participants. Secondly, our data are from an English population, so it is unclear whether our findings would be

For peer review only - http://bmjopen.bmj.com/site/about/guidelines.xhtml

For peer review only

generalizable to the rest of the United Kingdom. The STS does not provide us data on co-morbidity or have any follow-up data on mortality. It is therefore possible that GPs choose not to try to change smoking behaviours in those patients who have multiple morbidities or a poor life expectancy, which may well be clinically appropriate.

Finally, our analyses are based on respondents and, while we have no data on non-response bias, it is reasonable to assume that those who chose not to participate in the voluntary STS are more likely to be older those who did respond are probably healthier than the general older population. Whilst this may limit generalizability, it is likely that this sub-sample is of greater clinical interest as they are more likely to benefit from stopping smoking than frailer individuals with potentially more limited life expectancy.

Implications for research, policy and practice

It is unclear from our findings what proportion of smokers who were not offered interventions to stop smoking may have benefitted from them and would have wished to consider these options. Clearly smokers are under no obligation to try and quit but we suspect in some cases this may be due to not understanding the potential benefits or assuming there are none. Qualitative research could help to gain greater insight into why older patients and their GPs do not pursue smoking cessation, so that appropriate interventions can be designed to reduce inequitable access. For example, recent research has demonstrated that the use of targeted invitations to stop smoking services increased attendance in all age groups, including people aged 65+, with the relative benefits larger in this age band than younger groups. [33] This supports our view that it may be easier to change behaviour in this group if motivated..Older adults need to be offered smoking cessation interventions that are acceptable, appropriate and available, even if they decide not to use them. With the re-commissioning of smoking cessation services, there is an opportunity to ensure that currently underserved groups are supported in attempts to stop smoking or reducing their intake. Evaluative studies are required to determine the most cost-effective type, location and mode of delivery of these interventions to ensure that older smokers can also realise the health benefits of quitting into their 60s and beyond.

Conclusions

This large national study has demonstrated that older smokers report being less likely to quit or reduce consumption, though are less nicotine dependent. Potential opportunities to

For peer review only - http://bmjopen.bmj.com/site/about/guidelines.xhtml

facilitate cessation are being missed as older smokers report that GPs are less likely to offer interventions or specific advice, though this may reflect past patient preferences. Service provision should consider how best to reduce these variations and a stronger effectiveness evidence base is required to support commissioning for this older population.

Acknowledgements

We thank Jamie Brown and Robert West of the Clinical, Educational & Health Psychology, Division of Psychology & Language Sciences, Faculty of Brain Sciences, University College London for their support with accessing the Smoking Toolkit Study. The Smoking Toolkit Study is currently primarily funded by Cancer Research UK (C1417/A14135; C36048/A11654; C44576/A19501), and has previously also been funded by Pfizer, GlaxoSmithKline, and the Department of Health. JB's post is funded by a fellowship from the Society for the Study of Addiction, and Cancer Research UK also provide support (C1417/A14135).

Competing interests

The authors declare they have no competing interests

Funding

This work was undertaken as part of the Ageing Well programme, funded by the NIHR School for Public Health Research (SPHR). The views expressed are those of the author(s) and not necessarily those of the NHS, the NIHR or the Department of Health.

Data sharing statement

The data that support these findings are available from the Smoking Toolkit Study. Restrictions apply to the availability of these data, which were used under licence for the current study, and so are not publicly available. Data are however available from the authors on reasonable request and with the permission of Robert West at robertwest100@googlemail.com

Contributor statement

The study was initially conceived and designed by YB-S, HJ and MH, with contributions from all authors. Data analysis was carried out by MH and YB-S.

For peer review only - http://bmjopen.bmj.com/site/about/guidelines.xhtml

All authors contributed to drafting and critically revising the paper.

All authors have approved the final version and are equally accountable for the work.

References

1. ASH. Smoking statistics. 2016.
2. Taylor DH, Hasselblad V, Henley SJ, et al. Benefits of smoking cessation for longevity. Am J Public Health. 2002;92(6):990–6.
3. Østbye T, Taylor DH. Smoking and Substance Abuse The Effect of Smoking on Years of Healthy Life (YHL) Lost among Middle-Aged and Older Americans. Health Serv Res. 2004;39(3):531–52.
4. US Department of Health and Human Services. The health benefits of smoking cessation. A report of the Surgeon General. 1990;627.
5. NICE. Stop smoking services. 2008.
6. Huddlestone L, Walker GM, Hussain-Mills R, Ratschen E. Treating tobacco dependence in older adults: a survey of primary care clinicians' knowledge, attitudes and practice. BMC Family Practice. 2015; 16:97 .
7. Cawkwell PB, Blaum C, Sherman SE. Pharmacological Smoking Cessation Therapies in Older Adults : A Review of the Evidence. Drugs Aging. Springer International Publishing; 2015;32(6):443–51. doi:10.1007/s40266-015-0274-9
8. ASH Scotland. Tobacco and Older Adults: a literature review. Edinburgh: ASH Scotland 2004.
9. Kerr S, Watson H, Tolson D, Lough M, Brown M. Smoking after the age of 65 years: a qualitative exploration of older current and former smokers' views on smoking, stopping smoking, and smoking cessation resources and services. Health Soc Care Community. 2006;14:572–82.
10. Twigg L, Moon G, Szatkowski L, Iggulden P. Smoking cessation in England: intentionality, anticipated ease of quitting and advice provision. Soc Sci Med. 2009; doi: 10.1016/j.socscimed.2008.11.032
11. Maguire CP, Ryan J, Kelly a, O'Neill D, Coakley D, Walsh JB. Do patient age and medical condition influence medical advice to stop smoking? Age Ageing 2000;29(3):264–6.
12. Huang Y, Britton J, Hubbard R, Lewis S. Who receives prescriptions for smoking cessation medications? An association rule mining analysis using a large primary care database. Tobacco Control 2013;22:274-279.

For peer review only - http://bmjopen.bmj.com/site/about/guidelines.xhtml

13. Wilson A, Hippisley-Cox J, Coupland C, Coleman T, Britton J, Barrett S. Smoking cessation treatment in primary care: prospective cohort study. Tobacco Control. 2005.14(4):242–6.

14. West R, May S, West M, Croghan E, McEwen A. Performance of English stop smoking services in first 10 years: analysis of service monitoring data BMJ 2013; 347 :f4921

15. Gilbert H, Sutton S, Sutherland G. Who calls QUIT? The characteristics of smokers seeking advice via a telephone helpline compared with smokers attending a clinic and those in the general population. Public Health. 2005;119(10):933–9.

16. von Elm E, Altman DG, Egger M, Pocock SJ, Gotzsche PC, Vandenbroucke JP. The Strengthening the Reporting of Observational Studies in Epidemiology (STROBE) Statement: guidelines for reporting observational studies. BMJ. 2007;335(7624):806-808

17. Fidler J A, Shahab L, West O, Jarvis MJ, McEwen A, Stapleton J a, et al. "The smoking toolkit study": a national study of smoking and smoking cessation in England. BMC Public Health. 2011;11(1):479.

18. Heatherton TF, Kozlowski LT, Frecker RC, Rickert W, Robinson D. Measuring the Heaviness of Smoking: using self-reported time to the first cigarette of the day and number of cigarettes smoked per day. Addiction. 1989;84(7):791–800.

19. Heatherton TF, Kozlowski LT, Frecker RC, FargerstromKarl-Olov. The Fagerström test for nicotine dependence: a revision of the Fagerstrom Tolerance Questionnaire. Br J Addict. 1991;86(9):1119–27.

20. National Readership Survey. Social Grade. http://www.nrs.co.uk/nrs-print/lifestyle-and-classification-data/social-grade. Accessed 01 Oct 2016.

21. Hyland A, Borland R, Li Q, Yong H-H, McNeill A, Fong GT, et al. Individual-level predictors of cessation behaviours among participants in the International Tobacco Control (ITC) Four Country Survey. Tob Control. 2006;15 Suppl 3:iii83–94.

22. Lang I A, Rice NE, Wallace RB, Guralnik JM, Melzer D. Smoking cessation and transition into retirement: analyses from the English Longitudinal Study of Ageing. Age Ageing. 2007;36(6):638–43.

23. Zantinge E, Vn den Berg M, Smit A, Picavet HSJ. Retirement and a healthy lifestyle : opportunity or pitfall ? A narrative review of the literature. Eur J Public Heal. 2013;24(3):433–9.

24. Jarvis MJ, Wardle J, Waller J, Owen L. Prevalence of hardcore smoking in England,

For peer review only - http://bmjopen.bmj.com/site/about/guidelines.xhtml

and associated attitudes and beliefs : cross sectional study. BMJ. 2003;326:1061.

25. Yong H, Borland R, Siahpush M. Quitting-related beliefs , intentions , and motivations of older smokers in four countries : findings from the international tobacco control policy evaluation survey. 2005;30:777–88.

26. Kotz D, Fidler J, West R. Factors associated with the use of aids to cessation in English smokers. Addiction. 2009;104(8):1403–10.

27. Zbikowski SM, Magnusson B, Pockey JR, Tindle HA, E WK. A review of smoking cessation interventions for smokers aged 50 and older. Maturitas. 2012;71:131–41.

28. Connolly M Smoking cessation in old age: closing the stable door? Age and Ageing. 2000;29:193-195

29. Steinberg MB, Akincigil A, Delnevo CD, Crystal S, Carson JL. Gender and age disparities for smoking-cessation treatment. Am J Prev Med. 2006;30(5):405–12.

30. Chen D, Wu L-T. Smoking cessation interventions for adults aged 50 or older: a systematic review and meta analysis. Drug Alcohol Depend. 2015; 154:14-24.

31. Zhu S, Melcer T, Sun J, Rosbrook B, Pierce JP. Smoking Cessation With and Without Assistance A Population-Based Analysis. 2000;18(4):305–11.

32. Grundy E, Holt G. The socioeconomic status of older adults : How should we measure it in studies of health inequalities ? J Epidemiol Community Heal. 2001;55:895–904.

33. Gilbert H, Morris R et al. Effectiveness of personalised risk information and taster sessions to increase the uptake of smoking cessation services (Start2quit): a randomised controlled trial. The Lancet. 2017; 389:823-833.

For peer review only

For peer review only - http://bmjopen.bmj.com/site/about/guidelines.xhtml

Table 1: Association between socio-demographics and time period with smoking status and number of cigarettes consumed per day.

		Smoking Status								Cigarettes per day			
		Current vs. Never Smoker				Recent/Past vs. Never Smoker							
		OR	P-value	95% CI		OR	P-value	95% CI		OR*	P-value	95% CI	
Age													
	16-54	1.00				1.00				1.00			
	55-59	0.92	<0.001	0.88	0.97	1.79	<0.001	1.70	1.89	1.80	<0.001	1.68	1.93
	60-64	0.71	<0.001	0.68	0.75	2.19	<0.001	2.09	2.29	1.91	<0.001	1.79	2.04
	65-69	0.62	<0.001	0.59	0.66	2.65	<0.001	2.53	2.78	1.79	<0.001	1.66	1.92
	70-74	0.42	<0.001	0.39	0.44	2.41	<0.001	2.30	2.53	1.51	<0.001	1.39	1.64
	75-79	0.29	<0.001	0.27	0.31	2.35	<0.001	2.23	2.48	1.18	0.001	1.07	1.30
	80-84	0.21	<0.001	0.19	0.23	2.44	<0.001	2.29	2.60	0.94	0.330	0.82	1.07
	85+	0.13	<0.001	0.12	0.15	2.36	<0.001	2.19	2.54	0.73	0.001	0.61	0.89
	p-value for trend		<0.001				<0.001				<0.001		
Gender													
	Men	1.34	<0.001	1.31	1.37	1.39	<0.001	1.35	1.43	1.49	<0.000	1.44	1.54
	Women	1.00				1.00				1.00			
Social Grade													
	AB	1.00				1.00				1.00			
	C1	1.56	<0.001	1.50	1.63	1.00	0.897	0.97	1.04	1.03	0.416	0.96	1.09
	C2	2.40	<0.001	2.31	2.50	1.09	<0.001	1.05	1.14	1.31	<0.001	1.23	1.39
	D	2.88	<0.001	2.76	3.00	0.97	0.156	0.92	1.01	1.31	<0.001	1.23	1.40
	E	4.72	<0.001	4.52	4.92	0.96	0.078	0.92	1.00	1.62	<0.001	1.53	1.72
	p-value for trend		<0.001				0.008				<0.001		
Survey Years													
	2006-08	1.00				1.00				1.00			
	2009-10	0.88	<0.001	0.86	0.91	0.90	<0.001	0.86	0.94	0.86	<0.001	0.82	0.90
	2011-12	0.88	<0.001	0.86	0.91	1.14	<0.001	1.10	1.19	0.64	<0.001	0.61	0.68
	2013-15	0.80	<0.001	0.77	0.83	1.26	<0.001	1.22	1.31	0.55	<0.001	0.52	0.58
	p-value for trend		<0.001				<0.001				<0.001		

* Ordered logistic regression with following outcome categories: 10 or fewer cigarettes per day, 11-20 cigarettes per day, and 21 or more cigarettes per day.

For peer review only

Table 2: Association between socio-demographics and time period with nicotine dependency and quit attempts over the past year

		High nicotine dependency					Any quit attempts in past year			
		OR	P-value	95% CI			OR	P-value	95% CI	
Age										
	16-54	1.00					1.00			
	55-59	1.35	<0.001	1.25	1.44		0.74	<0.001	0.68	0.80
	60-64	1.31	<0.001	1.22	1.40		0.70	<0.001	0.64	0.76
	65-69	1.17	<0.001	1.08	1.26		0.65	<0.001	0.59	0.71
	70-74	0.98	0.669	0.90	1.07		0.57	<0.001	0.51	0.64
	75-79	0.69	<0.001	0.62	0.76		0.41	<0.001	0.35	0.48
	80-84	0.54	<0.001	0.47	0.63		0.27	<0.001	0.21	0.35
	85+	0.45	<0.001	0.37	0.56		0.32	<0.001	0.22	0.46
	p-value for trend		<0.001					<0.001		
Gender										
	Men	1.21	<0.001	1.17	1.26		0.81	<0.001	0.78	0.85
	Women	1.00					1.00			
Social Grade										
	ab	1.00					1.00			
	C1	1.20	<0.001	1.12	1.28		0.96	0.228	0.89	1.03
	C2	1.61	<0.001	1.51	1.72		0.91	0.014	0.85	0.98
	d	1.89	<0.001	1.77	2.02		0.88	0.001	0.81	0.95
	e	2.45	<0.001	2.29	2.61		0.90	0.005	0.84	0.97
	p-value for trend		<0.001					<0.001		
Survey Years										
	2006-08	1.00					1.00			
	2009-10	0.95	0.033	0.91	1.00		0.80	<0.001	0.76	0.84
	2011-12	0.80	<0.001	0.76	0.85		0.73	<0.001	0.69	0.77
	2013-15	0.72	<0.001	0.68	0.76		0.85	<0.001	0.81	0.90
	p-value for trend		<0.001					<0.001		

For peer review only

Table 3: Association between socio-demographics and time period with discussion of smoking at GP consultation and whether the doctor offered any active management.

For peer review only

For peer review only - http://bmjopen.bmj.com/site/about/guidelines.xhtml

Box 1 Definitions of terms and outcomes

Nicotine dependency (Yes/No) Derived from Heaviness of Smoking Index (HSI) combining two scales (0-3) on "How soon after you wake up do you smoke your first cigarette?" (sooner gets higher score) and "How many cigarettes per day do you usually smoke?" (more cigarettes gets higher score). Scores from both questions are added for each respondent (min=0, max=6) and we dichotomized this variable into a high (3-6) and low (0-2) score.

Quit attempt (Yes/No) This was derived from the following question, "How many serious attempts to stop smoking have you made in the last 12 months?" asked to current smokers and recent smokers (in last 12 months). This was dichotomised as any serious attempts to stop smoking within the last year or no attempts.

Cutting down (Yes/No). This was only asked of current smokers and was coded from the question "Are you trying to cut down on how much you smoke?" (waves 1-32) which was later changed to "Are you currently trying to cut down on how much you smoke but not currently trying to stop?" (waves 33-66, 68, 70, 72, 74, 76, 78-102)

Use of NRTs (Yes/No) Respondents who were current smokers trying to cut down could report use of one or more of the following NRT products: Nicotine replacement gum, lozenges/tablets, inhaler, nasal spray, patch, or mouth spray. We dichotomised use of any NRT products versus none (question not included in waves 67, 69, 71, 73, 75, and 77).

Use of NHS advice services (Yes/No) Current smokers who responded that they had at least one serious quit attempt were shown a list of potential cessation services: NHS Stop Smoking Service group; one to one counselling/advice support session; contact with local NHS Stop Smoking Services; NHS smoking helpline; or Quitline. Use of NHS Smoke Free website was not included in this list because it was available to a small subset of the survey (waves 26-102).

Patient raised smoking (Yes/No) This was captured by a dichotomous variable to the question "Have you raised the topic of smoking with your GP in the past year (i.e. last 12 months)?" asked of current smokers and recent ex-smokers (quit within last 12 months).

Doctor raised smoking (Yes/No) This came from the question, "Has your GP spoken to you about smoking in the past year (i.e. last 12 months)?" This question was asked in waves 31-39 to current and former smokers (quit within last 12 months or more) and in waves 40-102 to current smokers and recent ex-smokers (quit within last 12 months).

Doctor management If Doctor raised smoking was YES then the following responses were available: (i) he/she suggested that I go to a specialist stop smoking advisor or group or that I see a nurse in the practice (**Counselling**); (ii) he/she offered me a prescription for a nicotine patch, nicotine gum, or other nicotine product (**NRT Prescription**); (iii) he/she suggested that I go to a specialist stop smoking advisor or group, or see a nurse in the practice and offered me a prescription for a nicotine patch, nicotine gum or another nicotine product (**Counselling and NRT prescription**); (iv) Yes, he/she advised me to stop but did not offer anything (**Advice only**); (v) Yes, he/she asked me about my smoking but did not advise me to stop smoking (**no active management**). These responses are available in waves 40-102 to current smokers and recent ex-smokers (quit within last 12 months).

For peer review only - http://bmjopen.bmj.com/site/about/guidelines.xhtml

Legend for Figures

Figure 1: Odds ratios for past use of nicotine replacement therapies (NRTs) and NHS stop smoking clinics by age group

Figure 2: Odds ratios for GP management (NRTs, counselling, both or no advice to quit) of smokers by age groups.

For peer review only

For peer review only - http://bmjopen.bmj.com/site/about/guidelines.xhtml

For peer review only

For peer review only - http://bmjopen.bmj.com/site/about/guidelines.xhtml

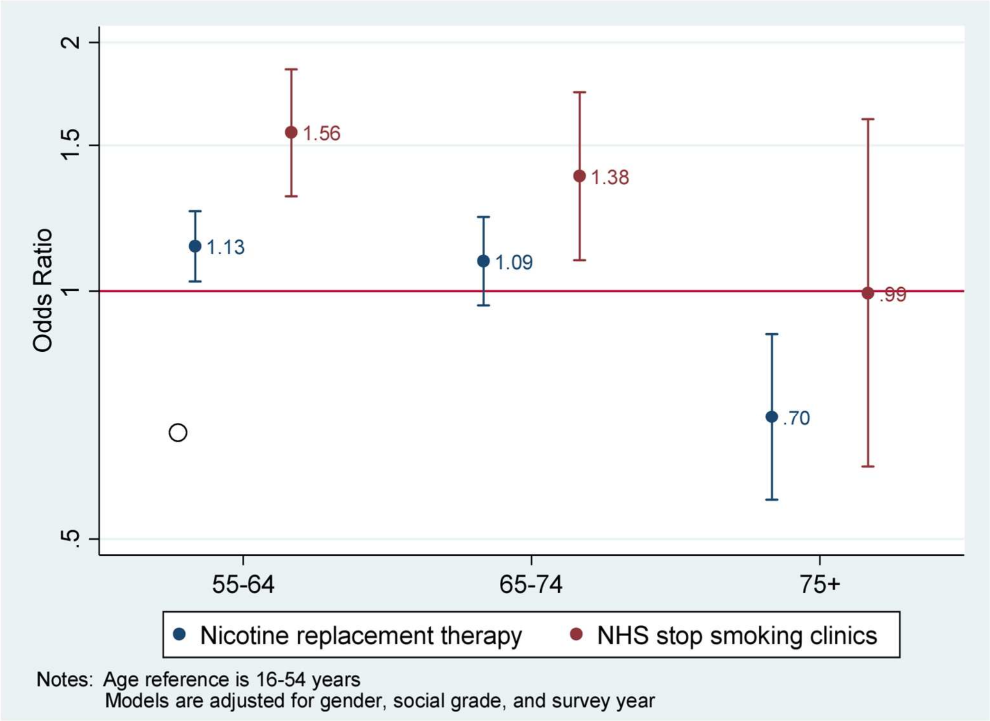

Caption : Figure 1: Odds ratios for past use of nicotine replacement therapies (NRTs) and NHS stop smoking clinics by age group

141x103mm (300 x 300 DPI)

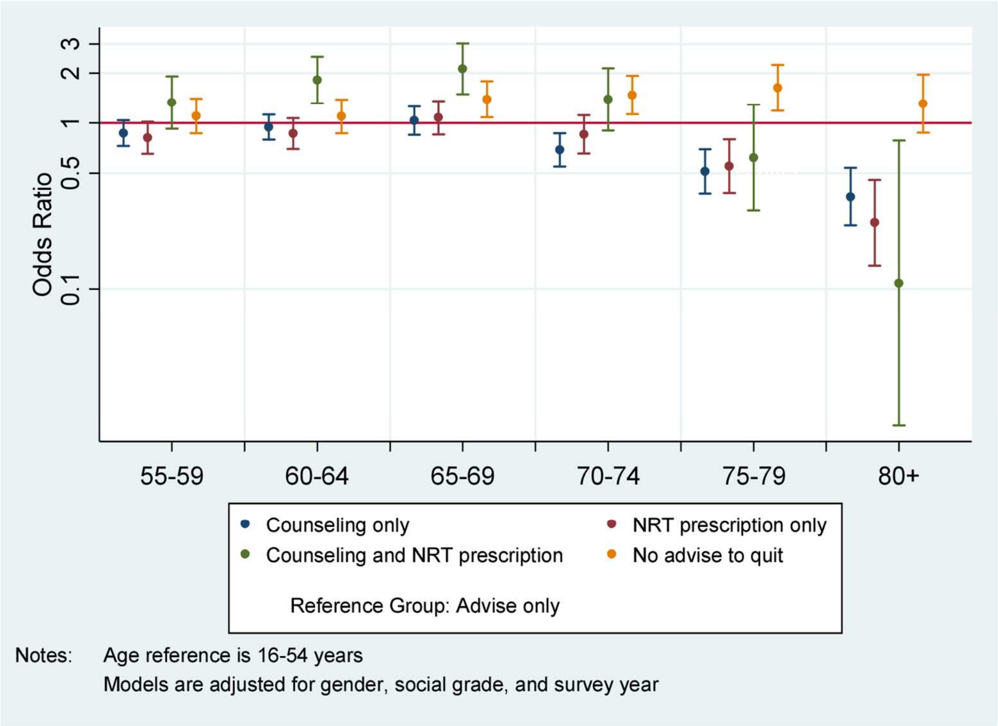

Caption : Caption : Figure 2: Odds ratios for GP management (NRTs, counselling, both or no advice to quit) of smokers by age groups.

140x102mm (300 x 300 DPI)

Supplemental Tables

Supplemental Table 1: Descriptive statistics of sociodemographics and time period with smoking status, past quitting and use of cessation therapies or GP management

		Ever Smoker	Nicotine Dependency	Quit attempts in past year	Past NRT use	Past NHS cessation clinics use	Respondent raised the topic of smoking with GP	GP spoke to respondent about quitting smoking	GP referrals to smoking cessation therapies
		N=68,967	N=51,920	N=43,385	N=20,286	N=16,026	N=11,432	N=29,578	N=10,466
**Age**		%	%	%	%	%	%	%	%
	**16-54**	61.4	69.4	74.6	75.8	80.1	73.6	73.7	69.2
	**55-59**	7.0	6.8	6.6	6.9	5.8	6.7	6.8	7.5
	**60-64**	8.2	7.2	6.4	6.4	5.4	6.4	6.6	7.6
	**65-69**	7.7	6.1	5.1	5.0	4.1	5.8	5.4	6.6
	**70-74**	6.2	4.5	3.6	3.1	2.6	3.6	3.8	4.6
	**75-79**	4.7	3.2	2.2	1.7	1.2	2.1	2.2	2.7
	**80-84**	3.0	1.8	1.1	0.7	0.4	1.0	1.1	1.2
	**85+**	1.9	0.9	0.5	0.3	0.2	0.7	0.6	0.7
	**Total**	100.0	100.0	100.0	100.0	100.0	100.0	100.0	100.0
**Gender**		%	%	%	%	%	%	%	%
	**Men**	48.0	51.0	50.5	48.4	47.2	54.1	52.0	47.4
	**Women**	52.0	49.0	49.5	51.6	52.8	46.0	48.0	52.7
	**Total**	100.0	100.0	100.0	100.0	100.0	100.0	100.0	100.0
**Social Grade**		%	%	%	%	%	%	%	%
	**AB**	15.2	12.4	10.5	10.6	10.9	10.8	10.6	10.5
	**C1**	24.9	23.8	22.9	22.9	23.7	24.8	22.8	21.8
	**C2**	22.2	22.5	22.7	22.6	22.7	23.7	22.4	21.5
	**D**	16.9	18.3	19.3	18.8	18.9	20.0	19.4	18.9
	**E**	20.8	23.1	24.6	25.1	23.8	20.7	24.8	27.3
	**Total**	100.0	100.0	100.0	100.0	100.0	100.0	100.0	100.0
**Survey Year**		%	%	%	%	%	%	%	%
	**2006**	2.1	2.8	2.3	2.6	2.8			
	**2007**	13.3	17.6	14.4	16.2	16.5			
	**2008**	9.9	13.1	11.1	12.4	11.8			
	**2009**	10.5	13.9	11.9	13.1	11.8		11.7	
	**2010**	13.7	13.2	14.0	14.6	13.4		20.2	21.9
	**2011**	12.5	10.2	12.2	12.6	10.8		17.1	19.0
	**2012**	11.9	9.9	11.9	8.1	10.8	12.0	17.0	20.0
	**2013**	12.7	9.3	10.7	9.2	11.0	42.2	16.3	18.4
	**2014**	10.8	8.2	9.4	9.2	9.4	37.1	14.3	16.7
	**2015**	2.8	1.9	2.2	2.0	1.8	8.7	3.3	4.1
	**Total**	100.0	100.0	100.0	100.0	100.0	100.0	100.0	100.0

For peer review only

Supplemental Table 2: Odds ratios for sociodemographics and time period with past use of NRTs or stop smoking counselling services

		**Past NRT use**					**Past NHS advice services use**			
		**OR**	**P-value**	**95% CI**			**OR**	**P-value**	**95% CI**	
**Age**										
	**16-54**	1					1			
	**55-64**	1.132	0.012	1.028	1.247		1.555	<0.001	1.303	1.856
	**65-74**	1.086	0.182	0.962	1.227		1.378	0.007	1.089	1.742
	**75+**	0.703	0.003	0.559	0.886		0.994	0.981	0.613	1.613
**Gender**										
	**Men**	0.903	0.003	0.845	0.966		0.773	<0.001	0.680	0.879
	**Women**	1					1			
**Social Grade**										
	**AB**	1					1			
	**C1**	0.884	0.047	0.783	0.999		0.920	0.478	0.731	1.158
	**C2**	0.887	0.054	0.785	1.002		0.890	0.327	0.706	1.123
	**D**	0.801	0.001	0.705	0.909		1.059	0.630	0.838	1.338
	**E**	0.899	0.081	0.797	1.013		0.973	0.815	0.776	1.221
**Survey Years**										
	**2006-08**	1					1			
	**2009-10**	1.065	0.138	0.980	1.158		0.885	0.122	0.758	1.033
	**2011-12**	0.839	<0.001	0.764	0.922		0.612	<0.001	0.512	0.733
	**2013-15**	0.597	<0.001	0.540	0.661		0.495	<0.001	0.409	0.599

For peer review only

Supplemental Table 3: Odds ratios for sociodemographics with different GP management compared to no referral

		GP referrals to help quit smoking															
		Counselling vs. No Referral				Pharmacology vs. No Referral				Counselling and Pharmacology vs. No Referral				GP did not advise to quit smoking vs. No Referral			
		OR	P-value	95% CI		OR	P-value	95% CI		OR	P-value	95% CI		OR	P-value	95% CI	
**Age**																	
	**16-54**	1				1				1				1			
	**55-59**	0.869	0.126	0.726	1.040	0.814	0.068	0.653	1.016	1.331	0.125	0.924	1.916	1.102	0.428	0.866	1.403
	**60-64**	0.944	0.515	0.794	1.123	0.864	0.179	0.697	1.069	1.821	<0.001	1.318	2.515	1.097	0.443	0.866	1.389
	**65-69**	1.034	0.734	0.851	1.258	1.077	0.530	0.855	1.356	2.127	<0.001	1.497	3.024	1.391	0.010	1.081	1.789
	**70-74**	0.690	0.002	0.547	0.869	0.854	0.244	0.654	1.114	1.390	0.137	0.900	2.146	1.476	0.004	1.128	1.930
	**75-79**	0.512	<0.001	0.377	0.696	0.551	0.002	0.380	0.799	0.618	0.201	0.296	1.291	1.636	0.002	1.190	2.248
	**80+**	0.361	<0.001	0.241	0.539	0.251	<0.001	0.138	0.456	0.109	0.028	0.015	0.787	1.309	0.191	0.875	1.959
**Gender**																	
	**Men**	0.820	<0.001	0.744	0.904	0.991	0.886	0.882	1.115	0.798	0.034	0.647	0.983	0.960	0.547	0.842	1.095
	**Women**	1				1				1				1			
**Social Grade**																	
	**AB**	1				1				1				1			
	**C1**	1.064	0.510	0.884	1.280	1.019	0.874	0.810	1.282	0.995	0.979	0.691	1.434	0.946	0.651	0.744	1.203
	**C2**	1.182	0.077	0.982	1.422	1.225	0.080	0.976	1.536	0.697	0.068	0.473	1.027	0.894	0.366	0.701	1.140
	**D**	1.129	0.208	0.935	1.363	1.184	0.153	0.939	1.491	0.728	0.112	0.492	1.077	0.908	0.444	0.708	1.163
	**E**	1.214	0.033	1.016	1.451	1.311	0.015	1.054	1.631	0.860	0.404	0.603	1.226	0.978	0.849	0.776	1.232
**Survey Years**																	
	**2009-10**	1				1				1				1			
	**2011-12**	0.899	0.101	0.792	1.021	1.325	<0.001	1.137	1.543	0.808	0.087	0.633	1.031	0.847	0.060	0.712	1.007
	**2013-15**	0.899	0.103	0.791	1.022	0.880	0.120	0.750	1.034	0.408	<0.001	0.307	0.540	0.983	0.844	0.829	1.166

For peer review only

Supplemental table 4: sensitivity analysis for association between age and whether the doctor raised smoking or offered any active management to quit

		GP offered any active management to quit								GP raised smoking							
		Original Model (sample from w40-102) (n=9,154)				Model with sample from w72-102 (n=3,800)				Original Model (sample from w40-102) (n=29,578)				Model with sample from w72-102 (n=11,425)			
		OR	P-value	95% CI		OR	P-value	95% CI		OR	P-value	95% CI		OR	P-value	95% CI	
Age																	
	16-54	1				1				1				1			
	55-59	1.14	0.126	0.97	1.34	1.06	0.675	0.82	1.36	1.58	0	1.44	1.74	1.65	0	1.43	1.93
	60-64	1.04	0.636	0.89	1.22	1.07	0.554	0.84	1.36	1.88	0	1.72	2.07	2.32	0	1.99	2.72
	65-69	0.90	0.233	0.75	1.07	0.85	0.208	0.66	1.10	1.98	0	1.78	2.19	2.38	0	2.02	2.80
	70-74	1.27	0	1.03	1.55	1.13	0.451	0.82	1.55	1.85	0	1.63	2.09	2.08	0	1.71	2.55
	75-79	1.87	0	1.43	2.44	1.82	0.006	1.19	2.79	1.69	0	1.44	1.98	1.78	0	1.37	2.30
	80+	3.16	0.002	2.20	1.55	2.34	0.002	1.38	3.98	1.02	0.804	0.85	1.24	1.30	0.08	0.97	1.74

For peer review only

For peer review only

STROBE 2007 (v4) Statement—Checklist of items that should be included in reports of *cross-sectional studies*

Section/Topic	Item #	Recommendation	Reported on page #
**Title and abstract**	1	(*a*) Indicate the study's design with a commonly used term in the title or the abstract	1
		(*b*) Provide in the abstract an informative and balanced summary of what was done and what was found	2-3
**Introduction**			
Background/rationale	2	Explain the scientific background and rationale for the investigation being reported	4
Objectives	3	State specific objectives, including any prespecified hypotheses	5
**Methods**			
Study design	4	Present key elements of study design early in the paper	5
Setting	5	Describe the setting, locations, and relevant dates, including periods of recruitment, exposure, follow-up, and data collection	5
Participants	6	(*a*) Give the eligibility criteria, and the sources and methods of selection of participants	5
Variables	7	Clearly define all outcomes, exposures, predictors, potential confounders, and effect modifiers. Give diagnostic criteria, if applicable	6-7
Data sources/ measurement	8*	For each variable of interest, give sources of data and details of methods of assessment (measurement). Describe comparability of assessment methods if there is more than one group	5
Bias	9	Describe any efforts to address potential sources of bias	5
Study size	10	Explain how the study size was arrived at	5
Quantitative variables	11	Explain how quantitative variables were handled in the analyses. If applicable, describe which groupings were chosen and why	6-7
Statistical methods	12	(*a*) Describe all statistical methods, including those used to control for confounding	7-8
		(*b*) Describe any methods used to examine subgroups and interactions	7
		(*c*) Explain how missing data were addressed	7-8
		(*d*) If applicable, describe analytical methods taking account of sampling strategy	-
		(*e*) Describe any sensitivity analyses	7-8
**Results**			

For peer review only

Participants	13*	(a) Report numbers of individuals at each stage of study—eg numbers potentially eligible, examined for eligibility, confirmed eligible, included in the study, completing follow-up, and analysed	8
		(b) Give reasons for non-participation at each stage	8
		(c) Consider use of a flow diagram	-
Descriptive data	14*	(a) Give characteristics of study participants (eg demographic, clinical, social) and information on exposures and potential confounders	8, Supplemental table 1
		(b) Indicate number of participants with missing data for each variable of interest	8
Outcome data	15*	Report numbers of outcome events or summary measures	8, Supplemental table 1
Main results	16	(*a*) Give unadjusted estimates and, if applicable, confounder-adjusted estimates and their precision (eg, 95% confidence interval). Make clear which confounders were adjusted for and why they were included	8, 9, Table1, Table 2, Table 3
		(*b*) Report category boundaries when continuous variables were categorized	Box 1
		(*c*) If relevant, consider translating estimates of relative risk into absolute risk for a meaningful time period	-
Other analyses	17	Report other analyses done—eg analyses of subgroups and interactions, and sensitivity analyses	10, Supplemental table 4
**Discussion**			
Key results	18	Summarise key results with reference to study objectives	10-11
Limitations	19	Discuss limitations of the study, taking into account sources of potential bias or imprecision. Discuss both direction and magnitude of any potential bias	11-12
Interpretation	20	Give a cautious overall interpretation of results considering objectives, limitations, multiplicity of analyses, results from similar studies, and other relevant evidence	11-12
Generalisability	21	Discuss the generalisability (external validity) of the study results	12
**Other information**			
Funding	22	Give the source of funding and the role of the funders for the present study and, if applicable, for the original study on which the present article is based	13

*Give information separately for cases and controls in case-control studies and, if applicable, for exposed and unexposed groups in cohort and cross-sectional studies.

Note: An Explanation and Elaboration article discusses each checklist item and gives methodological background and published examples of transparent reporting. The STROBE checklist is best used in conjunction with this article (freely available on the Web sites of PLoS Medicine at http://www.plosmedicine.org/, Annals of Internal Medicine at http://www.annals.org/, and Epidemiology at http://www.epidem.com/). Information on the STROBE Initiative is available at www.strobe-statement.org.

For peer review only

For peer review only
